# *In vivo* quantification of mitochondrial O_2_ affinity in human skeletal muscle using ^1^H‐magnetic resonance spectroscopy of deoxy‐myoglobin

**DOI:** 10.1113/JP290548

**Published:** 2026-05-24

**Authors:** Muhammet Enes Erol, Alexs A. Matias, Corinna F. Serviente, Stephen T. Decker, Rajakumar Nagarajan, Saadallah Ramadan, Yann Le Fur, Song‐Young Park, Gwenael Layec

**Affiliations:** ^1^ Department of Kinesiology and Institute for Applied Life Sciences University of Massachusetts Amherst Amherst Massachusetts USA; ^2^ School of Health and Kinesiology University of Nebraska Omaha Omaha Nebraska USA; ^3^ Department of Medicine University of Massachusetts Chan Medical School Worcester Massachusetts USA; ^4^ Center for Metabolic Health University of Utah Salt Lake City Utah USA; ^5^ Human Magnetic Resonance Center, Institute for Applied Life Sciences University of Massachusetts Amherst Amherst Massachusetts USA; ^6^ School of Health Sciences Faculty of Health and Medicine University of Newcastle Newcastle New South Wales Australia; ^7^ CRMBM Aix‐Marseille Université, CNRS Marseille France

**Keywords:** deoxymyoglobin ^1^H‐magnetic resonance spectroscopy, intracellular oxygen partial pressure, mitochondrial oxygen affinity, O_2_ cascade, skeletal muscle oxidative phosphorylation

## Abstract

**Abstract:**

Mitochondrial oxygen (O_2_) affinity is a fundamental determinant of oxidative phosphorylation capacity, which has yet to be directly measured in human skeletal muscle *in vivo*. To determine the apparent mitochondrial O_2_ affinity (i.e. *P*_50_) of the skeletal muscle, we used proton‐magnetic resonance spectroscopy (^1^H‐MRS) of deoxymyoglobin (dMb) during a circulatory occlusion of the lower limb to simultaneously quantify intracellular partial pressure of O_2_ (i $P_{O_{2}}$) and resting tissue‐specific O_2_ consumption (${\dot{V}}_{O_{2}}$) in the gastrocnemius muscle of sedentary young adults. Under these resting conditions, the ${\dot{V}}_{O_{2}}$–i $P_{O_{2}}$ relationship conformed to a Michaelis–Menten kinetics model (goodness of fit: *r* ^2^ = 0.84 ± 0.13). The estimated *V*_ss_, reflecting basal metabolic rate, reached 0.20 ± 0.06 mM min^−1^, and the apparent mitochondrial *P*_50_ was 0.50 ± 0.38 mmHg. *V*_ss_ and *P*_50_ were positively correlated (*r* = 0.85, *P* = 0.0009). This strong correlation remained after log transformation (*r* = 0.82, *P* = 0.0020). These results, obtained in human muscles *in vivo*, demonstrate that mitochondrial respiration exhibits a very high O_2_ affinity (*P*_50_ ≈ 0.5 mmHg). In addition, it quantitatively identifies the range across which oxidative phosphorylation in the skeletal muscle is O_2_‐independent (resting i $P_{O_{2}}$ under physiological conditions) or becomes O_2_‐sensitive (severe exercise and hypoxia). Moreover, the finding that muscle respiratory flux was associated with the apparent mitochondrial O_2_ affinity suggests additional regulatory mechanisms within the respiratory chain to fine‐tune oxidative phosphorylation to muscle ATP demand even at rest. This study provides a robust quantitative framework for interpreting *in vivo* respiratory control in both health and disease.

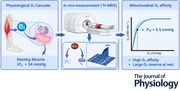

**Key points:**

- Mitochondrial oxygen affinity (*P*_50_) is central to oxidative phosphorylation, yet has not been directly quantified in human skeletal muscle *in vivo*.
- Using deoxymyoglobin ^1^H‐MRS during 8 min of lower‐limb ischaemia, we simultaneously measure intracellular partial pressure of O_2_ (i $P_{O_{2}}$) and resting tissue‐specific O_2_ consumption (${\dot{V}}_{O_{2}}$) in the resting gastrocnemius of healthy young adults.
- The ${\dot{V}}_{O_{2}}$–$P_{O_{2}}$ relationship followed Michaelis–Menten kinetics, yielding a *V*_ss_ of 0.20 mM min^−1^ and an apparent mitochondrial *P*_50_ of 0.50 mmHg, indicating very high O_2_ affinity.
- Resting apparent mitochondrial *P*_50_ was far below previously reported i $P_{O_{2}}$ (∼34 mmHg), indicating a wide O_2_‐independent range and identifying conditions (severe hypoxia, high‐intensity exercise) in which oxidative phosphorylation becomes O_2_‐sensitive.
- *V*_ss_ and *P*_50_ were positively correlated (*r* = 0.85), suggesting *in vivo* coupling between respiratory flux and oxygen affinity and providing a potential quantitative framework for interpreting mitochondrial respiratory control in health and disease.

## Graphical abstract

**Abstract figure legend** This graphical abstract illustrates a novel, non‐invasive approach to quantify mitochondrial oxygen (O_2_) affinity *in vivo* in human skeletal muscle using ^1^H magnetic resonance spectroscopy (^1^H‐MRS) of deoxymyoglobin (dMb). During brief arterial occlusion, dMb kinetics were used to simultaneously estimate intracellular oxygen tension (i $P_{O_{2}}$) and mitochondrial oxygen consumption (${\dot{V}}_{O_{2}}$) in the plantar flexor muscles. The relationship between ${\dot{V}}_{O_{2}}$ and i $P_{O_{2}}$ enabled the determination of the apparent mitochondrial *P*_50_ for O_2_, revealing a very high mitochondrial O_2_ affinity (*P*_50_ = 0.5 mmHg). These findings indicate that resting human skeletal muscle operates with a large O_2_ reserve, well above the range of mitochondrial O_2_ limitation.

## Introduction

The pathway for oxygen (O_2_) from the atmosphere to the mitochondria can be conceptually characterized as a series of transport systems involving the lungs, heart, circulation and skeletal muscle (Poole et al., 2022; Wagner, 1996; Weibel et al., 1991). As O_2_ moves through this pathway to sustain metabolic oxidation of substrates in cells, the partial pressure of O_2_ ($P_{O_{2}}$) declines at each step, from ∼150 mmHg in ambient air to ∼34 mmHg in skeletal muscle at rest (Richardson et al., 2006). Of note, such values represent typical approximations that can vary substantially depending on the environment and the metabolic state as the resting skeletal muscle only accounts for a modest fraction of whole‐body oxygen consumption. The final step of this conceptual cascade occurs in the myocyte, where O_2_ diffuses into the mitochondrial matrix and serves as the final electron acceptor during the process of oxidative phosphorylation (Mitchell, 1972). The fundamental importance of this process for cellular energy homeostasis is illustrated by the fact that oxidative phosphorylation accounts for up to 90% of the energy transduced under basal conditions in humans, resulting in the consumption of ∼350 L of O_2_ daily.

Given the critical importance of mitochondrial oxidative phosphorylation in the maintenance of homeostasis, determining the apparent oxygen affinity of mitochondrial respiration (*P*_50_) represents a major endeavour to better understand the regulation of oxidative metabolism in living tissues. However, due to the technical challenge in directly measuring intracellular O_2_ levels and mitochondrial O_2_ consumption, such quantification has been limited to *ex vivo* conditions using isolated cardiomyocytes and isolated mitochondria (Gnaiger et al., 1995; Gnaiger, Lassnig, Kuznetsov, Riger et al., 1998; Gnaiger, Lassnig, Kuznetsov & Margreiter, 1998; Gnaiger & Kuznetsov, 2002). With these preparations, the relationship between [O_2_] and mitochondrial respiration rate was fitted with a hyperbolic model (Michaelis–Menten kinetics) and an apparent *P*_50_, i.e. the half‐saturation point, ranging between 0.1 and 1.7 mmHg (Barzu & Satre, 1970; Cardinale et al., 2018; Gnaiger et al., 1995; Gnaiger, Lassnig, Kuznetsov & Margreiter, 1998; Larsen et al., 2011; St‐Pierre et al., 2000). In addition, this *P*_50_ was found to be modulated by several factors such as respiratory complex abundance and metabolic rate (Cardinale et al., 2018; Gnaiger, Lassnig, Kuznetsov, Riger et al., 1998; Larsen et al., 2011). However, those *ex vivo* preparations alter muscle fibre structural properties and O_2_ diffusion barriers, thus potentially biasing the sensitivity of the respiration rate to O_2_ (Picard et al., 2011; Richmond et al., 1999). These methodological considerations underscore the need to better characterize mitochondrial functional properties in the intracellular environment *in vivo*.

Interestingly, proton magnetic resonance spectroscopy (^1^H‐MRS) can detect myoglobin deoxygenation (dMb) *in vivo* in striated muscles (Kreutzer et al., 1992; Kreutzer & Jue, 1991; Wang et al., 1992). In perfused myocardium, Kreutzer & Jue (1991) demonstrated that the ^1^H‐resonance of the proximal histidine N‐delta (δ) proton of dMb appears in a spectrally isolated region and changes during hypoxic and ischaemic challenges. Because myoglobin is characterized by a single O_2_ binding site, the myoglobin–O_2_ association curve can be used to quantify the intracellular partial pressure of O_2_ (i $P_{O_{2}}$) in skeletal muscles (Lanza et al., 2010; Molé et al., 1999; Richardson et al., 1995). Both theoretical models and experimental evidence suggest that local O_2_ gradients around mitochondria are very small, implicating that cytosolic i $P_{O_{2}}$, measurable by ^1^H‐MRS, and intramitochondrial $P_{O_{2}}$ are similar *in vivo* (Clark et al., 1985, 1987). Another remarkable feature of dMb is that upon circulatory occlusion, its rate of change reflects muscle O_2_ consumption (Erol et al., 2025; Wang et al., 1990). This technique, therefore, offers the unique prospect of simultaneously quantifying skeletal muscle O_2_ consumption and i $P_{O_{2}}$ in the same tissue sample of skeletal muscle *in vivo*.

The goal of this study was thus to quantitatively determine mitochondrial respiration sensitivity for O_2_ *in vivo* by characterizing the relationship between intracellular $P_{O_{2}}$ and tissue‐specific respiration rate of the skeletal muscle using ^1^H‐MRS. We hypothesized that the ${\dot{V}}_{O_{2}}$–i $P_{O_{2}}$ relationship will conform to a Michaelis–Menten curve. In addition, consistent with an O_2_ reserve under normal physiological conditions, the apparent mitochondrial *P*_50_ estimated *in vivo* by dMb will be markedly lower than previously documented muscle i $P_{O_{2}}$ at rest (∼34 mmHg, Richardson et al., 2006).

## Methods

### Ethical approval

The study was approved by the Human Research Protection Program of the University of Massachusetts Amherst (IRB No. 1665). The study conformed to the standards set by the *Declaration of Helsinki*, except for the registration in a database. Upon comprehensive explanation of all procedures and potential risks through verbal and written communication, written informed consent was obtained from each participant before enrolment in the study.

### Participants

Following informed consent procedures, 11 healthy young adults volunteered to participate in this study. The participants were 18–29 years old and did not participate in regular physical activity beyond that required for activities of daily living. This was confirmed by a physical activity‐level questionnaire and a uniaxial accelerometer (GT3X, actigraph, Pensacola, FL, USA) instrumented on the non‐dominant wrist for 7 days. All participants were non‐smokers, free from overt diabetes and any known cardiovascular, peripheral vascular, neuromuscular or pulmonary diseases, and not taking any medications known to alter metabolism. For the female participants, all tests were conducted within the first 7 days of the early follicular phase. All experimental trials were performed in a thermoneutral environment at the same time of day, with the participants fasted overnight and having refrained from strenuous exercise (>24 h), consumption of caffeine and alcohol (>12 h), and vitamins (>72 h). On the day of the experimental visit, a fasting blood sample was collected for evaluation of a complete blood cell count and lipid panel (University Health Services).

### Experimental design

Participants reported to the laboratory on a single visit after blood sampling (complete blood cell count and comprehensive metabolic panel) and anthropometric measurements, and were then familiarized with the procedures. Each participant was positioned supine in a whole‐body Skyra 3T MR scanner (Skyra, Siemens Healthineers, Erlangen, Germany), with the right lower leg immobilized using straps across the shank and distal to the patella. A transmit–receive four‐channel phased array coil (366 × 174 mm) with linear polarized elements (Siemens) was wrapped around the gastrocnemius muscle at its maximum cross‐sectional area. A blood pressure cuff was placed on the thigh and connected to a rapid cuff inflator (Hokanson, Belleview, WA, USA) to occlude blood flow to the lower leg.

#### ^1^H‐MRS data collection and analysis

The N‐delta (δ) proton of the proximal histidine of F8 of myoglobin in the deoxygenated state was selectively excited using a Gaussian pulse (500 µs duration, 10 kHz carrier frequency offset) following the spectral approach originally established in perfused myocardium to isolate the dMb ^1^H signal as a marker of intracellular oxygenation (Kreutzer & Jue, 1991). ^1^H‐MRS spectra were acquired using the following parameters (TR = 15 ms, receiver bandwidth = 10 kHz, acquisition duration = 12 ms, nominal flip angle = 70°, 128 data points, 667 averages per spectrum, effective time resolution = 10 s). A three‐plane scout proton MR image was obtained to determine the proper position of the selected muscle with respect to the coil for localization purposes. Then, optimization of magnetic field homogeneity was performed using advanced manual localized volume shimming and manual shimming on the proton signal. After the shimming procedure, the full width at half maximum was 37.3 ± 5.4 for ^1^H, demonstrating excellent homogeneity in the volume of interest. During the ^1^H‐MRS measurement, the cuff was rapidly inflated to 250 mmHg for 8 min to induce arterial occlusion to the lower leg during which Mb‐derived ${\dot{V}}_{O_{2}}$ (mM min^−1^) was measured in parallel with intracellular $P_{O_{2}}$.

Due to baseline distortion, the quantification of dMb was performed by manual integration of the peak on CSIAPO (Le Fur et al., 2010). During a circulatory occlusion and subsequent ischaemia of the investigated limb, the O_2_ demand is matched by the intramuscular O_2_ store such that the rise in the ^1^H‐MRS Mb signal reflects the muscle O_2_ demand (${\dot{V}}_{O_{2}}$) according to:

$${\dot{V}}_{O_{2}}=\left[\mathrm{Mb}\right]\;\cdot \;\mathrm{dM}b_{\mathrm{sat}}/dt$$

where dMb_sat_ represents Mb saturation and [Mb] represents total Mb content, which was assumed to be 0.5 mM in the skeletal muscle (Gros et al., 2010). The fraction of dMb was determined by normalizing signal areas to the average signal acquired during the 7th and 8th minute of ischaemia at the end of the protocol, as previously described by our laboratory (Erol et al., 2025). Mb‐derived ${\dot{V}}_{O_{2}}$ was calculated from the initial portion of the dMb desaturation time course (typically within the first ∼4–5 min of occlusions), whereas the full 8 min period was used to reach the end‐ischaemic plateau for normalization. Intracellular $P_{O_{2}}$ was simultaneously computed from the myoglobin oxygen‐binding curve according to:

$$P_{O_{2}}=\left[\frac{1\;-\;f}{f}\right]\;\cdot \;P_{50}$$

where 1 − ƒ is the fraction of myoglobin that is oxygenated, ƒ is the fraction of myoglobin that is not oxygenated and *P*_50_ is the O_2_ pressure where half of the myoglobin binding sites are bound to O_2_. The temperature‐dependent myoglobin *P*_50_ of 2.2 mmHg was used (Schenkman et al., 1997) based on an approximate muscle temperature of ∼36°C (Kenny et al., 2003). To derive the apparent mitochondrial *P*_50_ from the ${\dot{V}}_{O_{2}}$ *versus* $P_{O_{2}}$ relationship, a Michaelis–Menten kinetics model was used:

$${\dot{V}}_{O_{2}}=\frac{V_{\mathrm{ss}\;}\cdot P_{O_{2}}\;}{P_{O_{2}}+P_{50}}$$

where ${\dot{V}}_{O_{2}}$ denotes the measured ${\dot{V}}_{O_{2}}$ in the skeletal muscle, *V*_ss_ is the steady‐state in O_2_ consumption rate estimated from the ${\dot{V}}_{O_{2}}$–i $P_{O_{2}}$ fit under resting conditions, and *P*_50_ is the partial pressure of O_2_ at which O_2_ consumption reaches half of *V*_ss_.

### Statistical analysis

All statistical analyses were performed using GraphPad Prism (Version 9.4.1; San Diego, CA, USA) with significance accepted at *P* < 0.05. Data were linearly interpolated to be time‐aligned for the model fitting. For each participant, non‐linear least‐squares fitting was performed using a hyperbolic model to estimate *V*_ss_ and *P*_50_, analogous to Michaelis–Menten kinetics. Goodness‐of‐fit was evaluated via the coefficient of determination (*R* ^2^). Individual confidence intervals for each parameter were derived from the fit, and group‐level results are presented as mean ± SD.

## Results

### Participant characteristics

Characteristics of the participants are presented in Table 1. All 11 healthy young adults (6 males, 5 females, age: 22 ± 3 years) completed the study. The mean body mass index (BMI) of participants ranged from 17.5 to 29.1 kg m^−2^, with a group mean of 23.6 ± 3.6 kg m^−2^. Participants demonstrated low to moderate daily physical activity (7984 ± 2434 steps day^−1^) with a mean of 128 ± 40 min day^−1^ spent in moderate‐to‐vigorous intensity activity (MVPA). Fasting blood and plasma measures were all within the clinically acceptable reference ranges for healthy individuals, with no out‐of‐range abnormalities noted in white blood cell count, red blood cell volume or haemoglobin/haematocrit levels.

**Table 1:** Participant characteristics

Sample size (male/female)		11(6/5)	
	Reference values	Results	Range
**Anthropometric characteristics**			
Age (years)		22 ± 3.32	18–29
Height (cm)		172.9 ± 9.7	163–192
Weight (kg)		70.5 ± 12.8	91.3–51.1
BMI (kg m^−2^)		23.6 ± 3.6	17.5–29.1
**Physical activity**			
Step counts (count day^−1^)		7983.9 ± 2434.1	4302–13,812
MVPA (min day^−1^)		127.5 ± 40.1	60–218
**Blood and plasma characteristics**			
Glucose (mg dL^−1^)	70–99	87.3 ± 3.7	80–93
Cholesterol (mg dL^−1^)	≤200	176.4 ± 33. 8	116–225
Triglycerides (mg dL^−1^)	≤150	72.30 ± 18.5	40–95
HDL‐C (mg dL^−1^)	40–60	59.20 ± 15.1	42–86
LDL‐C (mg dL^−1^)	<160	102.6 ± 22.7	55–125
WBC (10^3^ µL^−1^)	4.8–10.8	5.7 ± 1.7	3.6–8.3
RBC (10^6^ µL^−1^)	4.7–6.1	4.8 ± 0.7	4.1–6.2
Haemoglobin (g dL^−1^)	14–18	14.3 ± 1.8	12.3–17.3
Haematocrit (%)	42–52	41.9 ± 4.8	37–50
Neutrophil no. (10^3^ µL^−1^)	1.40–6.50	3.1 ± 0.9	1.6–4.5
Lymphocyte no. (10^3^ µL^−1^)	1.20–3.40	2.1 ± 0.7	1.1–3
Monocyte no. (10^3^ µL^−1^)	0.1–0.6	0.5 ± 0.2	0.2–0.7

*Notes*: values are expressed as mean ± SD, units in parentheses (*n* = 11).

Abbreviations: BMI, body mass index; HDL, high density lipoprotein cholesterol; LDL, low density lipoprotein cholesterol; MVPA, moderate–vigorous physical activity; RBC, red blood cells; WBC, white blood cells.

### Myoglobin deoxygenation during ischaemia

**Figure 1:**
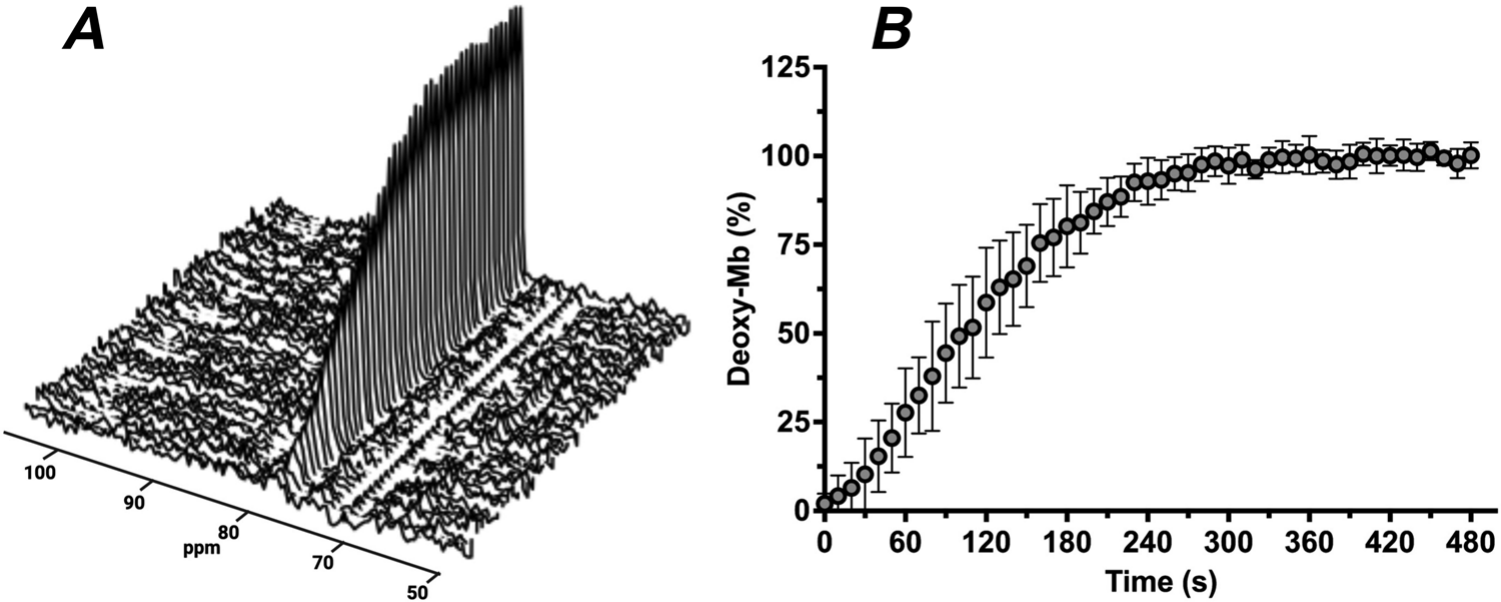
Myoglobin deoxygenation during lower limb ischaemia in young adults *A*, representative ^1^H‐MRS time series of spectra from a single participant during 8 min of arterial occlusion. *B*, group‐averaged time course of dMb at baseline and during 8 min of arterial occlusion. Values were normalized to each participant's end‐ischaemic plateau (100%) and shown as mean ± SD, *n* = 11. dMb rapidly increased from zero at baseline, reaching a steady‐state by ∼240 s that persisted throughout the remaining 480 s.

Following rapid arterial occlusion, the dMb signal rose progressively and reached a plateau within ∼4–5 min, remaining stable for the rest of the 8 min ischaemic period (Fig. 1). Figure 1*A* illustrates the ^1^H‐MRS time series of spectra from a representative participant in which the dMb resonance emerges immediately upon occlusion and rapidly grows during the ischaemia before reaching a steady‐state after a few minutes. Figure 1*B* indicates the group average dMb time course (*n* = 11), normalized to each participant's end‐ischaemic value, demonstrating the rapid rise and subsequent steady‐state.

### Mitochondrial O_2_ affinity and resting O_2_ consumption rate

**Figure 2:**
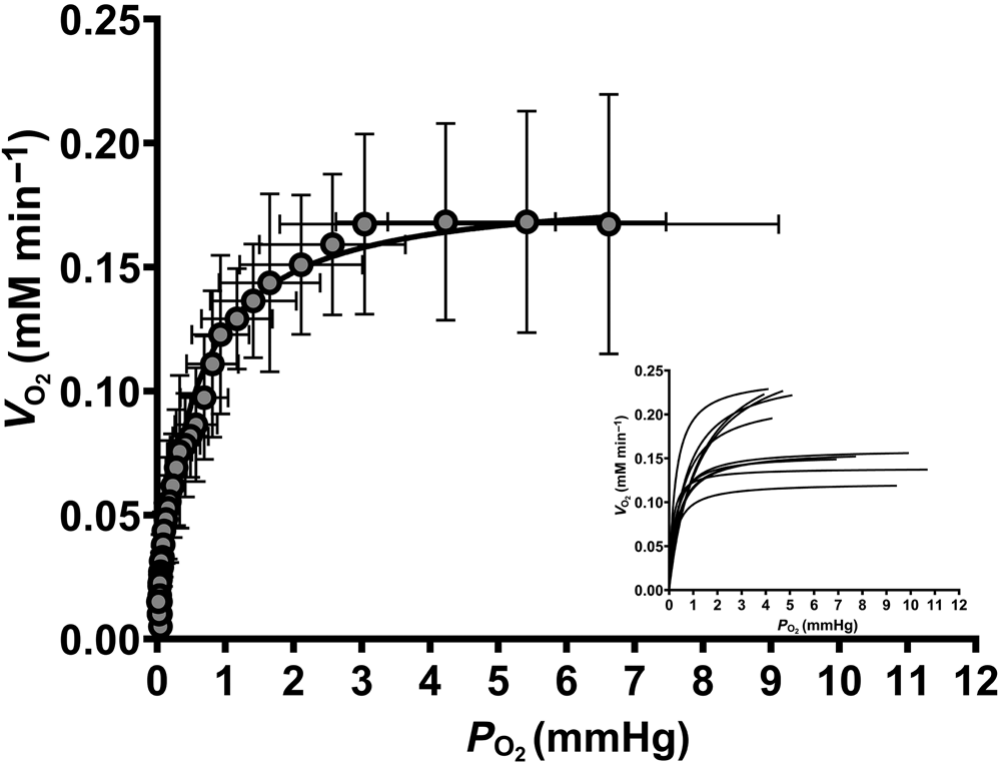
Relationship between intracellular oxygen partial pressure ($P_{O_{2}}$) and mitochondrial oxygen consumption (${\dot{V}}_{O_{2}}$) in the lower limb of young adults measured by ^1^H‐MRS of deoxymyoglobin The main panel shows the group‐averaged ${\dot{V}}_{O_{2}}$–$P_{O_{2}}$ relationship fitted with a Michaelis–Menten model to estimate mitochondrial *V*_ss_ (basal metabolic rate) and *P*_50_ (i.e. $P_{O_{2}}$ at half‐maximal ${\dot{V}}_{O_{2}}$), yielding a mean *V*_ss_ of 0.20 ± 0.06 mM min^−1^ and a mean *P*_50_ of 0.50 ± 0.38 mmHg. Values are shown as mean ± SD, with the individual fits plotted in the insets (*n* = 11).

**Figure 3:**
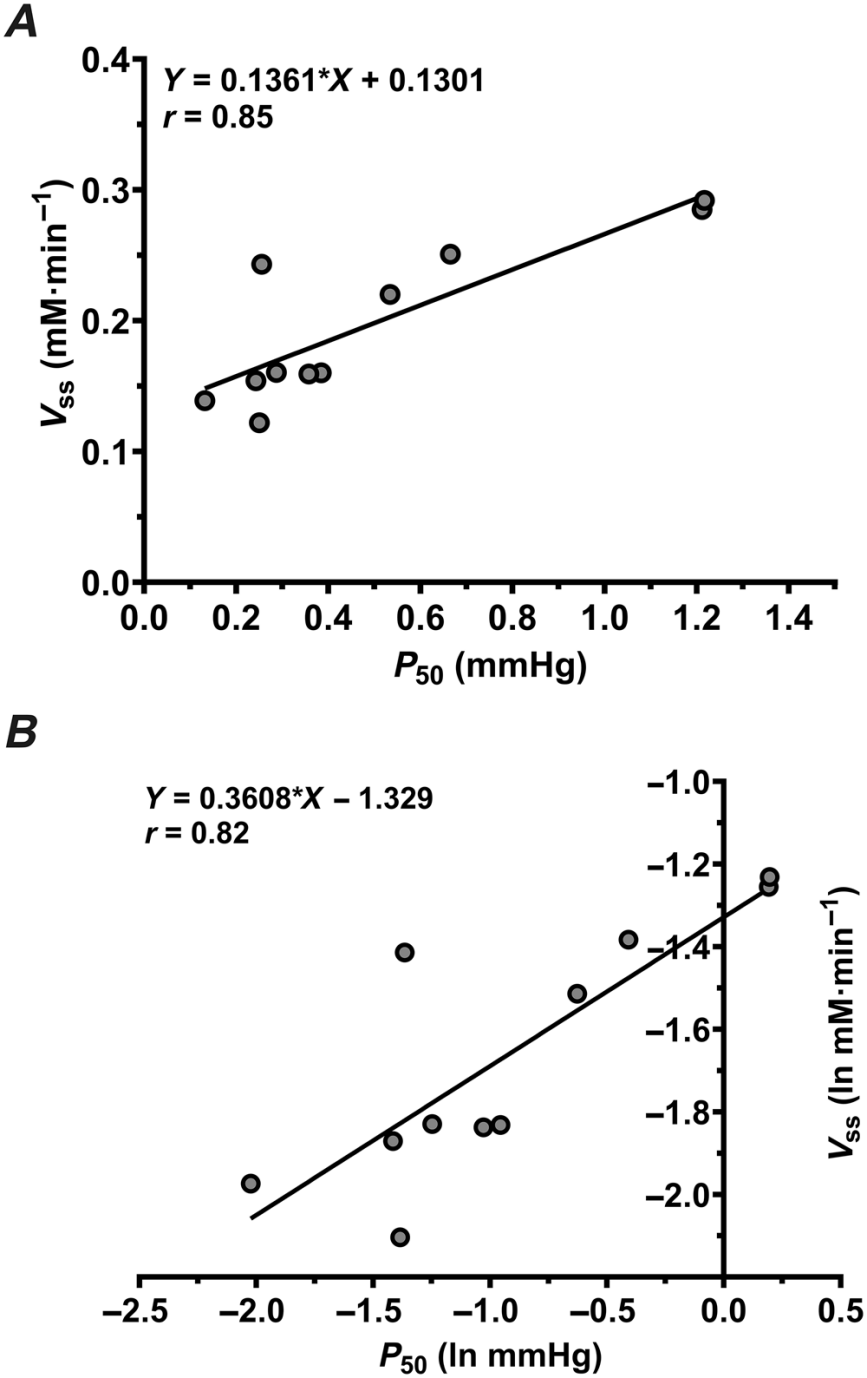
Relationship between steady‐state oxygen consumption rate (*V*_ss_) and apparent oxygen affinity (*P*_50_) in the lower limb of young adults *in vivo* *V*_ss_ and *P*_50_ were significantly and positively correlated (*r* = 0.85, *P* = 0.0009, *n* = 11, *A*). The association remained significant after log‐transformation (*r* = 0.82, *P* = 0.0020, *B*).

Using a Michaelis–Menten kinetics model to describe the relationship between tissue‐specific ${\dot{V}}_{O_{2}}$ and intracellular $P_{O_{2}}$, the apparent mitochondrial *P*_50_ (i.e. the $P_{O_{2}}$ at half‐maximal O_2_ consumption) and *V*_ss_ (i.e. ${\dot{V}}_{O_{2}}$ plateau) were derived for each participant (Fig. 2). *V*_ss_ was 0.20 ± 0.06 mM min^−1^ with a group mean of 95% CI of 0.17 ± 0.05 to 0.23 ± 0.09 mM min^−1^. The apparent *P*_50_ was 0.50 ± 0.38 mmHg with a group mean of 95% CI of 0.29 ± 0.21 to 0.65 ± 0.49 mmHg. The goodness of the fit was confirmed across participants with an average coefficient of determination (*r* ^2^) of 0.84 ± 0.13. Individual fits demonstrated typical hyperbolic relationships between ${\dot{V}}_{O_{2}}$ and $P_{O_{2}}$, supporting the use of the Michaelis–Menten kinetics model under ischaemic conditions. *V*_ss_ was positively correlated with *P*_50_ (*r* = 0.85; 95% CI, 0.51–0.96; *P* = 0.0009; Fig. 3). To confirm the robustness of this correlation, the analysis was repeated using log‐transformed variables, which confirmed a significant positive association between *V*_ss_ and *P*_50_: log‐transformed, *r* = 0.82 (95% CI 0.43–0.95), *P* = 0.002, *n* = 11 (slope = 0.361 in log units).

## Discussion

The goal of the present study was to quantitatively assess the O_2_ dependence of mitochondrial respiration *in vivo* in the plantar flexor muscles at rest using non‐invasive ^1^H‐MRS in humans. Using a state‐of‐the‐art methodology *in vivo*, this study provided several novel insights concerning the control of oxidative phosphorylation in the skeletal muscle: (1) the relationship between intracellular $P_{O_{2}}$ and dMb‐derived ${\dot{V}}_{O_{2}}$ conformed to a Michaelis–Menten kinetics model, indicating that oxidative phosphorylation was sensitive to O_2_ levels across the measured range (0–15 mmHg); (2) in resting skeletal muscle, the apparent mitochondrial *P*_50_ for O_2_ was extremely low (∼0.50 mmHg); and (3) the estimated oxidative phosphorylation *V*_ss_ under this condition was correlated to mitochondrial *P*_50_ values (*r* = 0.85) indicating an interaction between mitochondrial O_2_ affinity and mitochondrial oxidative phosphorylation metabolic rate at rest. The estimated *P*_50_ of ∼0.50 mmHg in the resting skeletal muscle supports the concept that mitochondrial oxidative phosphorylation exhibits an extremely high oxygen affinity.

### Estimation of the apparent mitochondrial *P*_50_ *in vivo versus ex vivo*

The simultaneous *in vivo* quantification of intracellular $P_{O_{2}}$ and ${\dot{V}}_{O_{2}}$ in the resting skeletal muscle with preserved muscle fibres and mitochondrial structural properties afforded by ^1^H‐MRS of dMb is a major strength of the present study. Using this state‐of‐the‐art technique, a central finding was that within intact human skeletal muscle, the relationship between intracellular $P_{O_{2}}$ and mitochondrial ${\dot{V}}_{O_{2}}$ followed the pattern of a Michaelis–Menten curve (Fig. 2), with mitochondrial respiration exhibiting measurable oxygen‐dependent kinetics across physiologically relevant intracellular $P_{O_{2}}$ ranges (<15 mmHg). Specifically, quantitative analysis of the i $P_{O_{2}}$–${\dot{V}}_{O_{2}}$ relationship revealed that the apparent mitochondrial *P*_50_ for O_2_ in resting human muscles was ∼0.50 mmHg *in vivo*. This value is akin to the estimated mitochondrial *P*_50_ from cell suspension (∼1.0 ± 0.62 mmHg; range 0.5–1.7 mmHg) (Robiolio et al., 1989; Rumsey et al., 1990; Wilson et al., 1977) and isolated mitochondria (0.50 ± 0.41 mmHg; range 0.11–1.65 mmHg) (Barzu & Satre, 1970; Cardinale et al., 2018; Gnaiger et al., 1995; Gnaiger, Lassnig, Kuznetsov & Margreiter et al., 1998; Larsen et al., 2011; St‐Pierre et al., 2000), as summarized in Fig. 3. Those values were computed from experiments in which respiration was stimulated by maximal ADP concentrations with Complex I and/or Complex II specific substrates, i.e. coupled respiration similar to the present conditions.

Of note, most *ex vivo* estimates reported here were obtained from closed‐chamber respirometers, enabling more accurate assessment of mitochondrial respiration than two‐phase system instruments, for which more heterogeneous $P_{O_{2}}$ introduces some errors in the estimation of the *P*_50_ at low $P_{O_{2}}$ in the early studies on this topic (Cole et al., 1982; Gnaiger et al., 1995). However, closed‐chamber respirometers still inherently exhibit O_2_ gradients, especially at higher respiratory flux (Donnelly et al., 2022), and various oxygenation strategies have been used across studies to attenuate such effects. For instance, cell‐suspension *P*_50_ estimates were obtained in sealed cuvette/syringe preparations, in which $P_{O_{2}}$ was allowed to decline progressively near air saturation (Robiolio et al., 1989; Rumsey et al., 1990; Wilson et al., 1977). For isolated mitochondria, measurements were generally conducted at ambient or sub‐ambient oxygenation (Barzu & Satre, 1970; St‐Pierre et al., 2000), except for the study by Cardinale et al. (2018), which hyperoxygenated the chambers. Despite this methodological difference, the resulting *P*_50_ values remained within a tight range, underscoring the high intrinsic O_2_ affinity of cytochrome c oxidase (COX). Overall, the majority of the literature, including our study, supports an apparent mitochondrial *P*_50_ for O_2_ above 0.1 and below 1.7 mmHg across *in vitro* and *in vivo* preparations, which also supports the concept that intramyocellular $P_{O_{2}}$ around the mitochondrial network is homogeneous at rest (Clark et al., 1987).

Somewhat in contrast to these results, Gayeski et al. (1987) previously estimated an apparent *P*_50_ of ∼0.06 mmHg in a canine gracilis *in situ* preparation. Using cryomicrospectroscopy during twitch contractions approaching maximal metabolic rates, the study concluded that ${\dot{V}}_{O_{2}}$ would decline only when intracellular $P_{O_{2}}$ approached ∼0.5 mmHg, implying an apparent Michaelis constant on the order of ∼0.06 mmHg (Gayeski et al., 1987). While methodological issues (poor technique sensitivity, heterogeneous sampling) question the validity of this value, this discrepancy can also be partly explained by physiological factors, including the metabolic state (resting *vs*. ${\dot{V}}_{O_{2}\mathrm{peak}}$) and differences in mitochondrial content (mixed muscle *vs*. predominantly oxidative muscle), both of which modulate the respiratory control and the O_2_ dependence of mitochondrial respiration.

Because both O_2_ distribution and mitochondrial density/morphology vary substantially within the muscle and across metabolically active organs (liver, heart, etc.), the mitochondrial *P*_50_ for O_2_ may also differ across tissues (Kietzmann, 2019; Willingham et al., 2021). In this context, the low apparent mitochondrial *P*_50_ for O_2_ documented here would allow adequate electron flux through the respiratory chain in highly metabolically active organs, while providing an additional mechanism to fine‐tune mitochondrial energetic coupling through subtle variations in *P*_50_ across tissues and individuals (see section ‘Interaction between metabolic rate and mitochondrial *P*_50_’). Along those lines, *in vivo* evidence demonstrated that, for a given basal ATP synthesis rate, substrate oxidation through the Krebs cycle, as measured by ^13^C‐MRS, was higher in the skeletal muscle of endurance‐trained adults than their sedentary counterparts (Befroy et al., 2008). Similarly, proton leak‐dependent respiration stimulated by fatty acid oxidation was increased by endurance training in isolated mitochondria from skeletal muscle (Tonkonogi et al., 2000). Together, these findings suggest that modest alterations in the coupling of the respiratory chain to the proton motive force can occur, probably to prevent an excessive increase in mitochondrial membrane potential and reactive oxygen species formation. Several mechanisms, including proton leak and changes in mitochondrial *P*_50_ for O_2_, probably play a role in these thermodynamic adjustments of oxidative phosphorylation that appear to occur concomitantly with morphological changes in the mitochondrial network.

### Integrated perspective of muscle O_2_ exchange and O_2_ uptake

The apparent mitochondrial *P*_50_ of ∼0.50 mmHg estimated in the skeletal muscle *in vivo* (Fig. 3) was substantially lower than previously documented resting venous $P_{O_{2}}$ (∼35–40 mmHg) (Sutton et al., 1988; Richardson et al., 2006), an index of end‐capillary $P_{O_{2}}$ in humans (Wagner, 1996). The capillary–myocyte membrane is indeed considered the critical barrier to O_2_ flux in the periphery (Colburn et al., 2020). Similarly, intramyocellular $P_{O_{2}}$, measured directly by ^1^H‐MRS in the lower limb of healthy young adults, is sizably higher (∼34 mmHg; Richardson et al., 2006) than mitochondrial *P*_50_. Together, these results illustrate both the large capillary‐to‐mitochondria O_2_ pressure gradient, which drives O_2_ flux toward COX, along with the high intrinsic affinity of mitochondrial respiration for O_2_ (Fig. 5). As a result, mitochondrial resting metabolic rate in the skeletal muscle is largely independent of O_2_ levels under most *in vivo* conditions, including extreme environments such as moderate to high altitude.

While recognizing that other physiological factors need to be considered (endocrine, gastrointestinal and cardio‐pulmonary responses), the low *P*_50_ of the skeletal muscle at rest of untrained young adults living at sea level appears compatible with sustainable life at altitudes corresponding to the highest human habitation (4500–5000 m) without mitochondria‐specific adaptations. Using data from a simulation of the Mt Everest Ascent, it can be estimated that the venous $P_{O_{2}}$ of unacclimatized individuals would still be maintained to ∼29–30 mmHg at this altitude (Sutton et al., 1988). In support of this, when breathing hypoxic gas (10% O_2_) at sea level, a condition that replicates the inspired O_2_ partial pressure of high altitude (*P*_IO2_ at 4500 m ∼80 mmHg), the intracellular $P_{O_{2}}$ measured at rest by ^1^H‐MRS amounted to ∼23 mmHg in the quadriceps muscle of young adults (Richardson et al., 2006). Based on Fig. 2, an intracellular $P_{O_{2}}$ in the range of 20–25 mmHg would still fall in the O_2_‐independent portion of the relationship between mitochondrial respiration and intracellular $P_{O_{2}}$. Overall, these studies are consistent with a substantial O_2_ reserve in the skeletal muscle of healthy adults under resting conditions. Specifically, the large gap between resting intracellular $P_{O_{2}}$ (34 mmHg) and mitochondrial *P*_50_ illustrated in Fig. 5 suggests that oxidative phosphorylation operates independently from O_2_ limitation in most physiological conditions *in vivo*. In stark contrast, despite the high affinity of mitochondrial respiration for O_2_, high‐intensity exercise may pose a serious challenge for the maintenance of oxidative phosphorylation. O_2_ binding to COX may indeed exert appreciable control over ATP production at the low intracellular $P_{O_{2}}$ encountered in the exercising muscle *in vivo*. Specifically, in healthy adults, during high‐intensity exercise to exhaustion, venous and intracellular $P_{O_{2}}$ fell to critically low levels, ∼18 and ∼2–5 mmHg, respectively (Bendahan et al., 2017; Molé et al., 1999; Richardson et al., 1995, 1999). Consistent with these values, recent studies in animals have reported that the interstitial $P_{O_{2}}$ near the muscle fibre surface reached 10 mmHg during high‐intensity electrical stimulation of the spinotrapezius (Hirai et al., 2018). Interestingly, hypoxic exercise to exhaustion can further decrease venous $P_{O_{2}}$ and intracellular $P_{O_{2}}$ to 15 and ∼1 mmHg, respectively, in humans (Richardson et al., 1995, 1999). Under such conditions, and assuming that mitochondrial O_2_ affinity is not or is only minimally influenced by the metabolic rate, the intracellular $P_{O_{2}}$ would fall on the O_2_‐dependent portion of the relationship between mitochondrial respiration and intracellular $P_{O_{2}}$ (Fig. 2).

### Interaction between metabolic rate and mitochondrial *P*_50_

**Figure 4:**
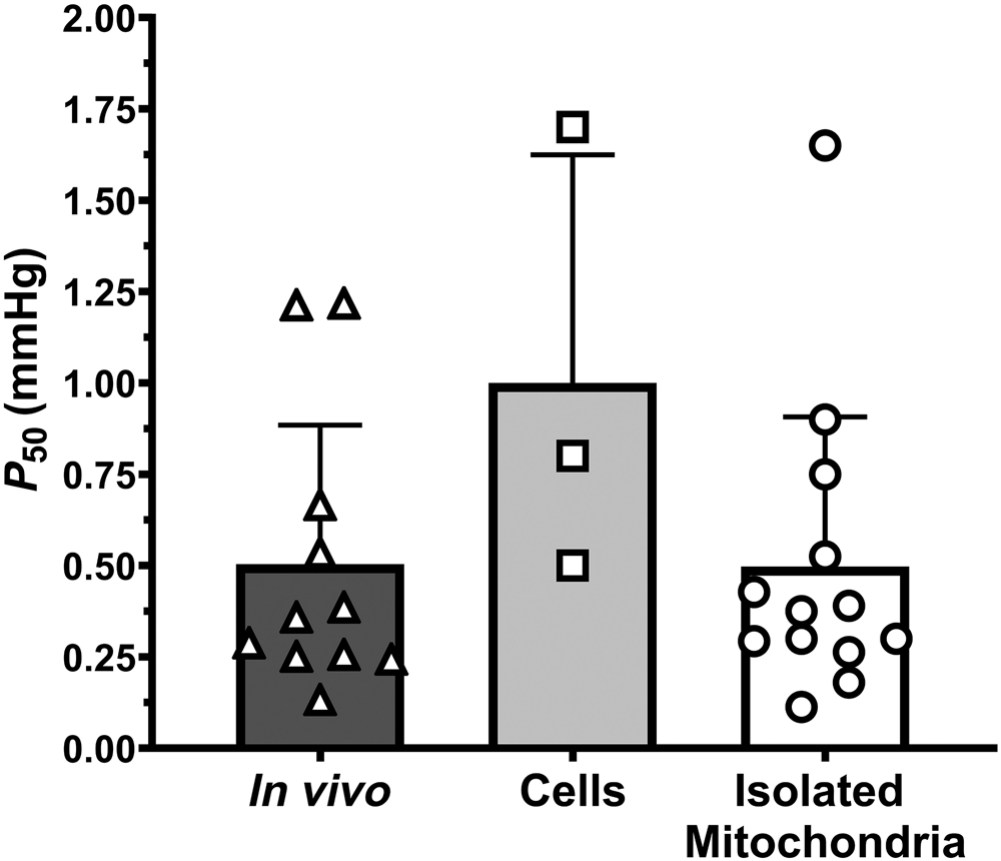
Apparent mitochondrial *P*_50_ for O_2_ *in vivo* and published values from *ex vivo* preparations Bars show the mean values, and black dots represent either the individual *P*_50_ estimates (present study, *n* = 11) or study‐specific average (cell suspension *n* = 3, isolated mitochondria *n* = 13). All *ex vivo* values were obtained with closed‐chamber respirometry at low $P_{O_{2}}$, in the range of 20–37°C, using either high‐resolution polarographic systems (Clark‐type/Ororboros O_2_k) or phosphorescence‐quenching oximetry, which enables continuous tracking of electrode‐measured chamber $P_{O_{2}}$ during controlled declines for *P*_50_ fitting. Isolated mitochondria were measured under ADP‐stimulated state‐3 with Complex I and/or Complex II specific substrates, while cell suspensions reflect coupled, quiescent conditions (high phosphorylation potential) (Barzu & Satre, 1970; Cardinale et al., 2018; Gnaiger et al., 1995; Gnaiger, Lassnig, Kuznetsov & Margreiter et al., 1998; Larsen et al., 2011; Robiolio et al., 1989; Rumsey et al., 1990; St‐Pierre et al., 2000; Wilson et al., 1977)

Another major finding of the present study was the positive correlation between *V*_ss_ and the mitochondrial *P*_50_ (*r* = 0.85, Fig. 4), indicating that individuals with higher resting metabolic rates concurrently exhibited lower mitochondrial oxygen affinity. This correlation suggests a trade‐off between respiratory rate and mitochondrial O_2_ affinity. Since these parameters were simultaneously measured *in vivo* under identical conditions, our findings provide robust evidence for a physiological coupling between mitochondrial respiratory rate and oxygen affinity. Consistent with this result, it is well documented in isolated mitochondria that *P*_50_ increases from the non‐phosphorylating state (leak respiration) to maximal ADP‐stimulated respiration (coupled respiration) (Gnaiger, Lassnig, Kuznetsov, Riger et al., 1998). Under basal conditions, only a fraction of the total cytochrome oxidase pool may be necessary for maintaining respiration, leaving substantial enzymatic reserve. As metabolic demand increases, this reserve is progressively utilized, thereby increasing per‐enzyme turnover and raising *P*_50_ (Gnaiger, Lassnig, Kuznetsov, Riger et al., 1998). In support of this interpretation, partial inhibition of COX by cyanide increased mitochondrial *P*_50_ without affecting maximal respiration rates in isolated mitochondria from the vastus lateralis of active young adults (Larsen et al., 2011).

Our observed correlation between higher basal metabolic rate and higher *P*_50_ *in vivo* may also reflect inter‐individual variation in metabolic control, driven by differences in muscle fibre type composition and/or mitochondrial density. Another important consideration is that secondary factors, such as levels of reactive oxygen species or nitric oxide (NO), which are both O_2_ dependent, may also fine‐tune intracellular O_2_ flux and mitochondrial O_2_ consumption to metabolic demand (Clanton, 2019; Taylor & Moncada, 2010). In the low‐$P_{O_{2}}$ operating range (Fig. 2), myoglobin (Mb) may influence intracellular $P_{O_{2}}$ dynamics beyond serving as an O_2_ transporter. Because Mb desaturates steeply as $P_{O_{2}}$ approaches its *P*_50_ (∼2.2 mmHg), it buffers rapid changes in intracellular $P_{O_{2}}$ and facilitates intracellular O_2_ transport when diffusion gradients increase at high O_2_ flux. Clanton (2019) conceptualized Mb as part of an integrated ‘power‐grid’ with the mitochondrial reticulum, proposing that Mb‐mediated buffering/facilitated diffusion (and $P_{O_{2}}$‐dependent NO interactions) can dampen intracellular $P_{O_{2}}$ heterogeneity and modulate respiratory control during heavy exercise. Future studies manipulating the redox balance or NO bioavailability are thus warranted to further evaluate the significance of this mechanism in modulating mitochondrial O_2_ affinity in the skeletal muscle *in vivo*.

### Mitochondrial *P*_50_ and metabolic control *in vivo*

**Figure 5:**
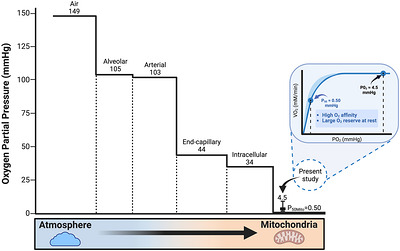
Conceptual illustration of the oxygen transport cascade from atmosphere to mitochondria, with mitochondrial *P*_50_ quantified *in vivo* by ^1^H‐MRS This schematic illustrates the progressive decline in oxygen partial pressure ($P_{O_{2}}$) along the oxygen cascade from atmospheric air (∼149 mmHg) to the mitochondrial level, adapted from Richardson et al. (2006). The present study extends this cascade by identifying mitochondrial oxygen affinity (*P*_50mito_; i.e. the $P_{O_{2}}$ at which mitochondrial respiration is 50% of maximal) *in vivo* at ∼0.50 mmHg in resting human skeletal muscle. An approximate $P_{O_{2}}$ value of ∼4.5 mmHg, derived from the fitted relationship, is shown and corresponds to ∼10% inhibition of respiration, illustrating the O_2_‐dependent portion of mitochondrial oxidative phosphorylation at rest. This value supports a steep $P_{O_{2}}$ gradient between the cytosol and mitochondria, highlighting the exceptionally high oxygen affinity of mitochondria in intact skeletal muscle of healthy young adults at sea level.

The estimated range of mitochondrial *P*_50_ (0.1–1.2 mmHg, Fig. 4) in the present study can also be integrated in the context of several models of respiratory control. According to the O_2_ cascade framework (Poole et al., 2021; Wagner, 1996; Weibel et al., 1991), a progressive decrement in $P_{O_{2}}$ from atmospheric air to the mitochondria provides effective O_2_ transport and cellular respiration *in vivo*. According to this theory, the large gap between resting intracellular $P_{O_{2}}$ (34 mmHg) and the mitochondrial *P*_50_ estimated in the present study (Fig. 5) allows both adequate O_2_ diffusion into the myocytes and oxidative phosphorylation to operate independently from O_2_ limitation in most *in vivo* conditions. A metabolic control analysis based on the range of mitochondrial *P*_50_ from the present study (0.1–1.2 mmHg) revealed that the control coefficient for O_2_ on the resting oxidative phosphorylation flux did not exceed 0.1 until i $P_{O_{2}}$ reaches ∼2 mmHg, thus quantitatively demonstrating that O_2_ did not exert much control over mitochondrial respiration *in vivo* under resting conditions. In other words, a given change in i $P_{O_{2}}$ above 2 mmHg affects by less than 10% the overall rate of oxidative phosphorylation, thus highlighting that the control of oxidative phosphorylation is distributed across multiple steps at rest.

The fundamental mechanisms by which oxidative ATP synthesis is controlled at rest and during muscle contraction are still a matter of debate (Korzeniewski & Rossiter, 2015; Wüst et al., 2011). However, key factors mediating the control of oxidative phosphorylation in the skeletal muscle are the feedback from the products of ATP hydrolysis (ADP and Pi) interacting with substrate‐level phosphorylation, mitochondrial redox potential (NADH/NAD^+^), O_2_ availability and mitochondrial enzymatic activities (Connett et al., 1990)_._ According to this concept, under conditions of low $P_{O_{2}}$, a greater increase in NADH, ADP and/or Pi would be required to maintain electron flux through the respiratory chain. This is supported by findings in contracting skeletal muscle in animals *in situ* and in humans *in vivo*, demonstrating that alterations in O_2_ availability induced by changes in inspired O_2_ fraction or ischaemia modulated the relationship between [Pi], [ADP], [PCr] or [ATP]/[ADP][Pi] and the rate of oxidative phosphorylation (Haseler et al., 1998; Hogan et al., 1999, 1992). Thus, while feedback by the energy state is crucial, the precise integration of signals (ADP, Pi, NADH/NAD^+^, Ca^2+^, etc.) is dynamic, and intracellular O_2_ availability is one important variable among these.

The apparent mitochondrial *P*_50_ for O_2_ measured *in vivo* can be further contextualized within the broader framework of oxygen uptake kinetics and metabolic transitions. During the transition from rest to constant‐load exercise, muscle ${\dot{V}}_{O_{2}}$ rises with a characteristic exponential time course that is commonly determined by either oxidative metabolic inertia within the contracting muscle or limitation in O_2_ supply to the active muscle fibres depending on cardiovascular fitness and the exercise intensity/modality (Hughson, 2009; Poole & Jones, 2012). In the moderate‐intensity domain, the major limitation to ${\dot{V}}_{O_{2}}$ kinetics originates from the delay in the activation of mitochondrial oxidative phosphorylation. However, as the metabolic rate increases and intracellular $P_{O_{2}}$ falls to critically low levels during repeated high‐intensity muscle contractions (<10 mmHg, Richardson et al., 1995, 2015), muscle ${\dot{V}}_{O_{2}}$ kinetics shifts toward an O_2_‐dependent limitation. Interestingly, the close correlation between *V*_ss_ and the mitochondrial *P*_50_ documented in the present study (Fig. 3) thus suggests the possibility of an additional interaction between mitochondrial affinity for O_2_ and the change in metabolic rate at the onset of exercise. Conceptually, this putative mechanism offers the advantage of mitigating intracellular O_2_ limitation while ensuring efficient energy transduction through oxidative phosphorylation.

### Experimental considerations

We conducted a secondary analysis to assess the potential influence of sex on the primary outcomes. This analysis did not reveal any appreciable sex differences within our small sample. Consistent with our secondary analysis, most studies have failed to detect differences in muscle metabolism and mitochondrial function. For instance, Kent‐Braun et al. (2002) found no significant sex‐related variation in muscle oxidative capacity assessed by PCr recovery kinetics in young adults, and Russ et al. (2005) similarly documented no sex differences in mitochondrial oxidative capacity as indicated by similar PCr recovery kinetics between men and women. Recently, Bailleul et al. (2025) reported no sex effect on fibre‐specific oxidative capacity. Overall, mitochondrial function thus appears mostly independent from sex differences.

## Conclusion

Using state‐of‐the‐art dMb ^1^H‐MR spectroscopy, we simultaneously measured intracellular $P_{O_{2}}$ and muscle‐specific ${\dot{V}}_{O_{2}}$ of the resting calf muscle in healthy adults. This *in vivo* approach demonstrated that the relationship between intracellular $P_{O_{2}}$ and the oxidative phosphorylation rate conformed to a Michaelis–Menten kinetic model with an apparent *P*_50_ of ∼0.50 mmHg. These results support the concept that mitochondrial oxidative phosphorylation exhibits an extremely high oxygen affinity in skeletal muscle and a substantial O_2_ reserve under resting conditions in healthy young, physically inactive adults. However, O_2_ levels may still exert some control over ATP production under specific conditions *in vivo* (e.g. severe hypoxia and high‐intensity exercise), unless adaptations in peripheral O_2_ supply or regulatory adjustments in mitochondrial oxidative phosphorylation occur. Interestingly, the finding that a greater respiratory flux was associated with a lower apparent mitochondrial affinity for O_2_ suggests the existence, *in vivo*, of additional regulatory mechanisms within the respiratory chain to fine‐tune oxidative phosphorylation to muscle ATP demand at rest. Overall, this study using a non‐invasive ^1^H‐MRS approach provides a quantitative framework for interpreting *in vivo* respiratory control in both health and disease.

## Additional information

## Competing interests

The authors declare no conflicts of interest.

## Author contributions

M.E.E. conceived and designed the work, acquired, analysed, and interpreted data, and drafted the manuscript. A.A.M. acquired and analysed data. C.F.S. acquired data. S.T.D. acquired data. R.N. conceived and designed the work and interpreted the results. S.R. conceived and designed the work and interpreted the results. Y.L.F. analysed data and edited and revised the manuscript. S.Y.P. provided input on the interpretation of the work. G.L. conceived and designed the work, analysed and interpreted data. All authors revised the manuscript critically for important intellectual content, approved the final version to be published, and agree to be accountable for all aspects of the work.

## Funding

This work was funded by the National Institute of Health Grant R01AG089307.

## Supporting information


Peer Review History

## Data Availability

The data of this study are available from the corresponding author upon reasonable request.
