# Supplementary material for: In vivo quantification of mitochondrial O2 affinity in human skeletal muscle using 1H‐magnetic resonance spectroscopy of deoxy‐myoglobin
Source: J Physiol. 2026 May 24;604(12):4876–88. doi: 10.1113/JP290548 (PMC13267685; doi:10.1113/JP290548)
Supplement: Supplementary file 1 — Peer Review History [file TJP-604-4876-s001.pdf]

The Journal of Physiology

In vivo Quantification of Mitochondrial O_2 Affinity in Human Skeletal Muscle Using ^{1}H-MR Spectroscopy of Deoxy-Myoglobin

Muhammet Enes Erol, Alexs A Matias, Corinna F Serviente, Stephen T Decker, Rajakumar Nagarajan, Saadallah Ramadan, Yann Le Fur, Song Young Park, and Gwenael Layec
DOI: 10.1113/JP290548

Corresponding author(s): Gwenael Layec (glayec@umass.edu)

The following individual(s) involved in review of this submission have agreed to reveal their identity: Robert Acton Jacobs (Referee #1); Kevin K McCully (Referee #2)

Review Timeline:

Submission Date:	14-Nov-2025
Editorial Decision:	12-Dec-2025
Revision Received:	10-Apr-2026
Accepted:	22-Apr-2026

Senior Editor: Karyn Hamilton

Reviewing Editor: Robert Musci

Transaction Report:

(Note: With the exception of the correction of typographical or spelling errors that could be a source of ambiguity, letters and reports are not edited. Depending on transfer agreements, referee reports obtained elsewhere may or may not be included in this compilation. Referee reports are anonymous unless the Referee chooses to sign their reports.)

Re: JP-RP-2025-290548 "**In vivo Quantification of Mitochondrial O_2 Affinity in Human Skeletal Muscle Using ^{1}H-MR Spectroscopy of Deoxy-Myoglobin**" by Muhammet Enes Erol, Alexs A Matias, Corinna F Serviente, Stephen T Decker, Rajakumar Nagarajan, Saadallah Ramadan, Yann Le Fur, Song Young Park, and Gwenael Layec

Dear Dr Layec,

Thank you for submitting your manuscript to The Journal of Physiology. It has been assessed by a Reviewing Editor and by 2 expert referees and we are pleased to tell you that it is potentially acceptable for publication following satisfactory major revision.

Please advise your co-authors of this decision as soon as possible.

The referee reports are copied at the end of this email.

Please address all the points raised and incorporate all requested revisions or explain in your Response to Referees why a change has not been made. We hope you will find the comments helpful and that you will be able to return your revised manuscript within 2 months. If your article is NOT for a Special Issue, you may have 9 months to revise. If you require an extension, please contact journal staff: jp@physoc.org. Please note that this letter does not constitute a guarantee for acceptance of your revised manuscript.

Your revised manuscript should be submitted online using the link in your Author Tasks: Link Not Available. This link is accessible via your account as Corresponding Author; it is not available to your co-authors. If this presents a problem, please contact journal staff (jp@physoc.org). Image files from the previous version are retained on the system. Please ensure you replace or remove any files that are being revised.

If you do not wish to submit a revised version of your manuscript, you must inform our journal staff (jp@physoc.org) or reply to this email to request withdrawal. Please note that a manuscript must be formally withdrawn from the peer review process at one journal before it may be submitted to another journal.

TRANSPARENT PEER REVIEW POLICY: To improve the transparency of its peer review process, The Journal of Physiology publishes online as supporting information the peer review history of all articles accepted for publication. Readers will have access to decision letters, including Editors' comments and referee reports, for each version of the manuscript, as well as any author responses to peer review comments. Referees can decide whether or not they wish to be named on the peer review history document.

ABSTRACT FIGURES: Authors are expected to use The Journal's premium BioRender account to create/redraw their Abstract Figures. Information on how to access this account is here: https://physoc.onlinelibrary.wiley.com/journal/14697793/biorender-access.

This will enable Authors to create and download high-resolution figures. If authors have used the free BioRender service, they can use the instructions provided in the link above to download a high-resolution version suitable for publication.

The link provided should only be used for the purposes of this submission. Authors will be charged for figures created on this account if they are not related to this manuscript submission.

LANGUAGE EDITING AND SUPPORT FOR PUBLICATION: If you would like help with English language editing, or other article preparation support, Wiley Editing Services offers expert help, including English Language Editing, as well as translation, manuscript formatting, and figure formatting at www.wileyauthors.com/eeo/preparation. You can also find resources for Preparing Your Article for general guidance about writing and preparing your manuscript at www.wileyauthors.com/eeo/prepresources.

RIGOUR AND REPRODUCIBILITY: We recommend authors consult The Journal's Rigour and Reproducibility requirements to ensure that their manuscript is suitable for publication: https://physoc.onlinelibrary.wiley.com/pb-assets/hub-assets/physoc/documents/TJP-Rigour-and-Reproducibility-Requirements.pdf

REVISION CHECKLIST:

Check that your Methods section conforms to journal policy: https://jp.msubmit.net/cgi-bin/main.plex?form_type=display_requirements#methods.

Check that data presented conforms to the statistics policy: https://jp.msubmit.net/cgi-bin/main.plex?form_type=display_requirements#statistics.

Upload a full Response to Referees file. To create your 'Response to Referees': copy all the reports, including any comments from the Senior and Reviewing Editors, into a Microsoft Word, or similar, file and respond to each point, using font or background colour to distinguish comments and responses and upload as the required file type.

Please upload two versions of your manuscript text: one with all relevant changes highlighted and one clean version with no changes tracked. The manuscript file should include all tables and figure legends, but each figure/graph should be uploaded as separate, high-resolution files.

You may also upload:

- 'Potential Cover Art' for consideration as the issue's cover image
- Appropriate Supporting Information (Video, audio or data set: see https://jp.msubmit.net/cgi-bin/main.plex?form_type=display_requirements#supp).

We look forward to receiving your revised submission.

If you have any queries, please reply to this email and we will be pleased to advise.

Yours sincerely,

Karyn Hamilton
Senior Editor
The Journal of Physiology

REQUIRED ITEMS FOR REVISION

- Author photo and profile. First or joint first authors are asked to provide a short biography (no more than 100 words for one author or 150 words in total for joint first authors) and a portrait photograph. These should be uploaded and clearly labelled together in a Word document with the revised version of the manuscript. See Information for Authors for further details.

- Please upload separate high-quality figure files via the submission form.

- Papers must comply with the Statistics Policy: https://jp.msubmit.net/cgi-bin/main.plex?form_type=display_requirements#statistics.

In summary:

- If n {less than or equal to} 30, all data points must be plotted in the figure in a way that reveals their range and distribution. A bar graph with data points overlaid, a box and whisker plot or a violin plot (preferably with data points included) are acceptable formats.

- If n > 30, then the entire raw dataset must be made available either as supporting information, or hosted on a not-for-profit repository, e.g. FigShare, with access details provided in the manuscript.

- 'n' clearly defined (e.g. x cells from y slices in z animals) in the Methods. Authors should be mindful of pseudoreplication.

- All relevant 'n' values must be clearly stated in the main text, figures and tables.

- The most appropriate summary statistic (e.g. mean or median and standard deviation) must be used. Standard Error of the Mean (SEM) alone is not permitted.

- Exact p values must be stated. Authors must not use 'greater than' or 'less than'. Exact p values must be stated to three significant figures even when 'no statistical significance' is claimed.

- Please include an Abstract Figure file and an Abstract Figure legend. An appropriate figure legend, which should not exceed 150 words in length, should be included in the main manuscript file. The Abstract Figure is a piece of artwork designed to give readers an immediate understanding of the research and should summarise the main conclusions. If possible, the image should be easily 'readable' from left to right or top to bottom. It should show the physiological relevance of the manuscript so readers can assess the importance and content of its findings. Abstract Figures should not merely recapitulate other figures in the manuscript. Please try to keep the diagram as simple as possible and without superfluous information that may distract from the main conclusion(s). Abstract Figures must be provided by authors no later than the

revised manuscript stage and should be uploaded as a separate file during online submission labelled as File Type 'Abstract Figure'. Please also ensure that you include the figure legend in the main article file. All Abstract Figures should be created using BioRender. Authors should use The Journal's premium BioRender account to export high-resolution images. Details on how to use and access the premium account are included as part of this email.

- Please ensure that all figures and tables have a title and legend, and that they have been cited within the main article text.

EDITOR COMMENTS

Reviewing Editor:

Thank you for your submission to Journal of Physiology. As you will find, both Referees found the manuscript very interesting and relevant. Both Referees have provided excellent feedback, all of which should be addressed.

Two particular comments standout:

While the conciseness of this manuscript is a strength, appropriately contextualizing the findings in the discussion is important. Please do consider Referee 1's suggestion to "situate your findings within these larger physiological frameworks."

Please also consider heeding Referee 2's comment to ensure that, where possible, use clear language and avoid jargon so as to broaden the accessibility of this manuscript. Please make explicit and clear(er) the theory and assumptions of this research.

Please also pay particular attention to comments and questions regarding assumptions in your modeling, these are also important to address.

Senior Editor:

Thank you for submitting your manuscript for consideration by The Journal of Physiology. As part of the peer review process, we recruited two Referees with expertise in this field of study. Both Referees were complimentary about the potential for impact if the concerns raised can be addressed with revisions. At this point, we would like to invite you to respond point-by-point to all concerns raised in the critiques, making corresponding revisions to the manuscript. We look forward to receiving your revised work and we thank you for your interest in The Journal of Physiology.

REFEREE COMMENTS

Referee #1:

The authors investigate mitochondrial oxygen affinity (P50) in human skeletal muscle using deoxymyoglobin ^{1}H-MRS during a controlled circulatory occlusion to simultaneously quantify intracellular PO_2 and resting VO_2. Their modeling of the VO_2-iPO_2 relationship yields estimates of Vmax and P50, which appear highly correlated, and they interpret these values as evidence of high mitochondrial O_2 affinity in vivo. The study aims to provide a quantitative basis for characterizing O_2-independent versus O_2-sensitive ranges of oxidative phosphorylation in resting skeletal muscle. While I find the study both interesting and valuable, I offer the following suggestions in the hope of further enhancing the clarity and impact of the

manuscript.

MAJOR

• The study design is intentionally clean and focused, which is a strength. However, this simplicity also limits the scope of the inferences that can be drawn with respect to whole-body oxygen kinetics and the broader regulation of oxidative phosphorylation. Enhancing the discussion by more explicitly situating your findings within these larger physiological frameworks would substantially strengthen the manuscript. A more fully developed biological context, linking your resting in vivo estimates to known principles of oxygen transport, mitochondrial control, and metabolic transitions, would greatly improve the interpretive depth and practical relevance of the study.

• One important consideration that appears to be missing is whether sex differed between the observed outcomes. While the sample size is admittedly small, reporting sex-specific descriptive data, effect sizes, and even low-powered comparisons would still provide valuable context, particularly in relation to research assessing for sex-specific differences in muscle metabolism and mitochondrial function. I also encourage the authors to clarify that the present manuscript represents the full analysis of these data, as it would not be appropriate for such a limited dataset to be partitioned into a separate publication focused solely on sex differences.

MINOR

Introduction:

• The opening paragraph provides a useful overview of the oxygen transport cascade; however, several aspects would benefit from clearer framing and greater physiological precision. As written, the paragraph presents specific partial pressure values (e.g., ambient, tissue, and intramuscular PO_2) and implies that O_2 transport and respiratory flux are occurring primarily in skeletal muscle. These assumptions should be stated more explicitly and contextualized, especially given that the present study investigates resting muscle, where skeletal muscle accounts for only a modest fraction of whole-body oxygen consumption. Clarifying that the cited PO_2 values represent typical approximations, rather than fixed physiological constants, would avoid unintentionally overgeneralizing conditions that vary with environment, measurement technique, and metabolic state. In addition, tightening the biochemical language would improve accuracy. For example, mitochondria do not "produce" energy; rather, they transduce the free energy from substrate oxidation into ATP through oxidative phosphorylation. Revising the phrasing accordingly (e.g., referring specifically to ATP production or energy transduction) would align the introduction with contemporary biochemical understanding. Overall, refining the assumptions and terminology in this initial paragraph will bring it into better alignment with the scientific precision demonstrated throughout the remainder of the Intro.

Results:

• For both Vmax and P50, the presentation of the results would benefit from clearer statistical formatting or explanation. The manuscript reports values such as "Vmax = 0.20 {plus minus} 0.06 mM·min^{-1} with the 95% CI of 0.17 {plus minus} 0.05 to 0.23 {plus minus} 0.09 mM·min^{-1}" and "P50 = 0.74 {plus minus} 0.55 mmHg with the 95% CI of 0.42 {plus minus} 0.30 to 0.87 {plus minus} 0.74 mmHg." As written, it is unclear whether the {plus minus} values accompanying the CI bounds represent inter-individual variability in the limits, or whether they denote another statistical quantity. Conventionally, the 95% CI for the group mean is expressed simply as two values (e.g., 0.17-0.23), whereas the SD or SEM is presented separately as mean {plus minus} SD (or SEM).

To improve clarity and interpretability, I suggest either adopting a more standard reporting structure (e.g., "Vmax = 0.20 {plus minus} 0.06 (mean {plus minus} SD), 95% CI: 0.17-0.23 mM·min^{-1}" and "P50 = 0.74 {plus minus} 0.55 (mean {plus minus} SD), 95% CI: 0.42-0.87 mmHg"), or adding a brief explanation describing precisely what the {plus minus} values around the CI limits represent. Either approach would help readers more easily understand the central tendency, variability, and precision of these kinetic estimates and avoid confusion about the meaning of the current presentation.

• Figure 2: It would be helpful to distinguish the VO_2-PO_2 curves for male and female participants in the lower right panel (e.g., by using solid vs. dashed lines) so that readers can more easily see whether sex-specific patterns are present. In addition, please confirm that all 11 participants are represented. The current panel appears to show 10 curves, although two may be superimposed.

• When referencing prior reports of P50 values from ex vivo preparations, could you please specify which studies employed hyperoxygenated chambers versus ambient (room air) saturation? This distinction is important for contextualizing differences in apparent oxygen affinity. You may also consider citing recent work from a lab I know you are familiar (PMID: 40803624), which discusses/details these importance of these methodological considerations.

Discussion:

• In the final sentence of the first discussion paragraph you state, "Yet, O_2 levels may still exert some control over ATP production under some specific conditions in vivo characterized by critically low intracellular PO_2." As currently placed, this assertion feels somewhat premature, as the manuscript does not experimentally examine the influence of altered PO_2 on respiratory flux or ATP production. I recommend removing this statement here and just focusing this discussion for later, as was included.

• Your comparison to ex vivo preparations requires some clarification. The manuscript suggests that 'closed-chamber respirometry minimizes O_2 gradients', but this is not consistent with established methodological work (Donnelly C, Schmitt S, Cecatto C, Cardoso LHD, Komlódi T, Place N, Kayser B, Gnaiger E (2022) The ABC of hypoxia - what is the norm. Bioenerg Commun 2022.12.v2. https://doi.org/10.26124/bec:2022-0012.v2). Closed-chamber systems inherently develop O_2 gradients, which is the very reason laboratories should employ hyperoxygenated environments (and why I am asking you to make this distinction across applicable references included throughout your paper). Hyperoxygenation mitigates, but does not eliminate, O_2 gradients, and it introduces its own considerations (e.g., increased ROS exposure). As currently written, the statement could inadvertently imply that closed-chamber designs reduce O_2-dependence without qualification, which may unintentionally validate suboptimal approaches in labs that do not hyperoxygenate. I suggest revising this portion of the discussion to more accurately reflect the limitations of closed-chamber respirometry or providing references to support the present interpretation.

• Paragraph 259-273: The section discussing how mitochondrial content may influence the observed Vmax and P50 values is particularly interesting, and I encourage the authors to expand this aspect of the discussion. A more concise summary of the Gayeski et al. (1987) findings would improve readability, while allowing more space to integrate your results with contemporary understanding of mitochondrial distribution/heterogeneity and respiratory control. Additionally, given that the current data are derived from a single muscle group at rest, it would be helpful to briefly elaborate on whether similar relationships would be expected in other skeletal muscles-or even in non-muscle tissues (i.e., liver, which can become much more hypoxic in specific areas than muscle) to further contextualize the broader relevance of your findings.

• Expanding the discussion to include the regulatory role of myoglobin in shaping intracellular PO_2 dynamics (e.g., PMID: 30335576) would add valuable biological context, particularly for interpreting how your resting measurements relate to conditions of increased metabolic demand, such as exercise. You reference this work toward the end of the discussion, which is an excellent start; extending this section slightly would help readers better understand how myoglobin-mediated buffering and facilitated diffusion influence mitochondrial PO_2 and respiratory control in vivo.

Referee #2:

This is an interesting paper that examines mitochondrial metabolism and oxygen affinity using 1H MRS. This reviewer has two general comments; which maybe related to an incomplete understanding of the theory and assumptions behind this study. I apologize if this comments have been addressed in this study or previous studies.

First, all of the results in the study appear to be calculated from the data shown in Figure 1B. If this is the case, wouldn't the correlations seen in Figure 4 be expected? Could the authors confirm the independence of their two main variables?

Second, the paper uses an assumed value of 0.5 mM for [Mb]. When calculating and comparing rate constants, it is not uncommon to assume a constant and uniform concentration. However, formally this leaves the resulting calculations with a maximum resolution of one significant digit. The importance of this assumption needs to be clarified in the discussion and limitations to the paper. As a suggestion, could the authors normalize their [Mb] values? Given the assumption that [ATP] values are tightly regulated, especially at rest, could the authors normalize their [Mb] values to [b-ATP] values? I am aware that 31P MRS was not performed in this study, but the authors should have this data from previous studies. Assuming a

mean value of 0.5 mM and normalizing might add precision to the measurements.

Minor comments

Vmax is used to represent the highest metabolic rate under resting conditions. Vmax is typically used to represent the highest enzymatic rate, and this is not achieved during resting conditions. This seems confusing and it would be helpful to the reader if a different terms was used.

Line 172. It seems confusing to refer to the 8 minute occlusion period. Mb VO2 was actually calculated from the first minute or second minute of the occlusion period?

Line 178. Minor point, but 39 degrees C is 102.2 Fahrenheit. Muscle temperature in the area under the MRS coil is most likely closer to 32-34 degrees C. (assuming skin is 30 degrees and the MRS data is colledted several cm deep into the muscle. This does not account for the wrap of the coil keeping the limb warm or the temperature of the room (which is often cool).

Figure 5. This is a nice graph. But there maybe some conceptual issues. Capillary values might be 'end capillary' as the fall in oxygen levels reflects the flux of oxygen from the vessels into the tissue. End capillary is after this exchange has occurred. Mitochondrial values might be listed in addition to the P50 values. Mitochondrial values might be expected to be higher than the P50. The P50 is not the actual oxygen pressures, but the value at which metabolism is half resting.

END OF COMMENTS

Reviewing Editor: Thank you for your submission to Journal of Physiology. As you will find, both Referees found the manuscript very interesting and relevant. Both Referees have provided excellent feedback, all of which should be addressed.

Two particular comments standout:

While the conciseness of this manuscript is a strength, appropriately contextualizing the findings in the discussion is important. Please do consider Referee 1's suggestion to "situate your findings within these larger physiological frameworks."

Please also consider heeding Referee 2's comment to ensure that, where possible, use clear language and avoid jargon so as to broaden the accessibility of this manuscript. Please make explicit and clear(er) the theory and assumptions of this research.

Please also pay particular attention to comments and questions regarding assumptions in your modeling, these are also important to address.

Author Response: Thank you for handling the review of our manuscript and the constructive feedback provided by both referees. We have carefully addressed all comments from Referee 1 and 2 in our point-by-point response below and revised the manuscript accordingly. Revisions include strengthening the broader physiological context in the Discussion, improving clarity and accessibility by reducing jargon, and making the underlying theory and modeling assumptions more explicit.

Senior Editor: Thank you for submitting your manuscript for consideration by The Journal of Physiology. As part of the peer review process, we recruited two Referees with expertise in this field of study. Both Referees were complimentary about the potential for impact if the concerns raised can be addressed with revisions. At this point, we would like to invite you to respond point-by-point to all concerns raised in the critiques, making corresponding revisions to the manuscript. We look forward to receiving your revised work and we thank you for your interest in The Journal of Physiology.

Reviewer #1:

The authors investigate mitochondrial oxygen affinity (P50) in human skeletal muscle using deoxymyoglobin ^{1}H-MRS during a controlled circulatory occlusion to simultaneously quantify intracellular PO_2 and resting VO_2. Their modeling of the VO_2-iPO_2 relationship yields estimates of Vmax and P50, which appear highly correlated, and they interpret these values as evidence of high mitochondrial O_2 affinity in vivo. The study aims to provide a quantitative basis for characterizing O_2-independent versus O_2-sensitive ranges of oxidative phosphorylation in resting skeletal muscle. While I find the study both interesting and valuable, I offer the following suggestions in the hope of further enhancing the clarity and impact of the manuscript.

The study design is intentionally clean and focused, which is a strength. However, this simplicity also limits the scope of the inferences that can be drawn with respect to whole-body oxygen kinetics and the broader regulation of oxidative phosphorylation. Enhancing the discussion by more explicitly situating your findings within these larger physiological frameworks would

substantially strengthen the manuscript. A more fully developed biological context, linking your resting in vivo estimates to known principles of oxygen transport, mitochondrial control, and metabolic transitions, would greatly improve the interpretive depth and practical relevance of the study.

Author Response: We thank the reviewer for their helpful comments and constructive feedback, which are both much appreciated. According to your suggestion, the discussion has been edited to provide additional physiological context, and we have expanded it to present our findings within the broader frameworks of oxygen transport, mitochondrial control, and metabolic transitions (Lines 375-419).

Mitochondrial P50 and metabolic control in vivo

The estimated range of mitochondrial P_{50} (0.1-1.2 mmHg, Figure 4) in the present study can also be integrated in the context of several models of respiratory control. According to the O_2 cascade framework (Poole et al., 2021; Wagner, 1996; Weibel et al., 1991), a progressive decrement in PO_2 from atmospheric air to the mitochondria provides effective O_2 transport and cellular respiration *in vivo.* According to this theory, the large gap between resting intracellular PO_2 (34 mmHg) and the mitochondrial P_{50} estimated in the present study (Figure 5) allows both adequate O_2 diffusion into the myocytes and oxidative phosphorylation to operate independently from O_2 limitation in most i*n vivo* conditions. A metabolic control analysis based on the range of mitochondrial P_{50} from the present study (0.1-1.2 mmHg) revealed that the control coefficient for O_2 on the resting oxidative phosphorylation flux did not exceed 0.1 until iPO_2 reaches ~2 mmHg thus quantitatively demonstrating that O_2 did not exert much control over mitochondrial respiration *in vivo* under resting conditions. In other words, a given change in iPO_2 affect by less than 10% the overall rate of oxidative phosphorylation thus highlighting that the control of oxidative phosphorylation is distributed across multiple steps.

The fundamental mechanisms by which oxidative ATP synthesis is controlled at rest and during muscle contraction are still a matter of debate (Korzeniewski & Rossiter, 2015; Wüst et al., 2011). However, key factors mediating the control of oxidative phosphorylation in the skeletal muscle are the feedback from the products of ATP hydrolysis (ADP and Pi) interacting with substrate-level phosphorylation, mitochondrial redox potential ($NADH/NAD^+$), O_2 availability, and mitochondrial enzymatic activities (Connett et al., 1990). According to this concept, under conditions of low PO_2 greater increase in NADH, ADP, and/or Pi would be required to maintain electron flux through the respiratory chain. This is supported by findings in contracting skeletal muscle in animals *in situ* and in humans *in vivo*, demonstrating that alterations in O_2 availability induce by changes in inspired O_2 fraction or ischemia modulated the relationship between [Pi], [ADP], [PCr] or [ATP]/[ADP][Pi] and the rate of oxidative phosphorylation (Haseler et al., 1998; Hogan et al., 1999; Hogan, et al., 1992). Thus, while feedback by the energy state is crucial, the precise integration of signals (ADP, P_i, $NADH/NAD^+$, Ca^{2+}, etc.) is dynamic, and intracellular O_2 availability is one important variable among these.

The apparent mitochondrial P_{50} for O_2 measured *in vivo* can be further contextualized within the broader framework of oxygen uptake kinetics and metabolic transitions. During the transition from rest to constant-load exercise, muscle VO_2 rises with a characteristic exponential time course that is commonly determined by either oxidative metabolic inertia within the contracting muscle or limitation in O_2 supply to the active muscle fibers depending on cardiovascular fitness and the exercise intensity/modality (Hughson, 2009; Poole & Jones, 2012).

In the moderate-intensity domain, the major limitation to VO_2 kinetics originates from the delay in the activation of mitochondrial oxidative phosphorylation. However, as the metabolic rate increases and intracellular PO_2 falls to critically low levels during repeated high-intensity muscle contractions (<10 mmHg, Richardson et al., 1995, 2015), muscle VO_2 kinetics shifts toward an O_2-dependent limitation. Interestingly, the close correlation between V_{ss} and the mitochondrial P_{50} documented in the present study (figure 4) thus suggests the possibility of an additional interaction between mitochondrial affinity for O_2 and the change in metabolic rate at the onset of exercise. Conceptually, this putative mechanism offers the advantage of mitigating intracellular O_2 limitation while ensuring efficient energy transduction through oxidative phosphorylation.

Reviewer 1: One important consideration that appears to be missing is whether sex differed between the observed outcomes. While the sample size is admittedly small, reporting sex-specific descriptive data, effect sizes, and even low-powered comparisons would still provide valuable context, particularly in relation to research assessing for sex-specific differences in muscle metabolism and mitochondrial function. I also encourage the authors to clarify that the present manuscript represents the full analysis of these data, as it would not be appropriate for such a limited dataset to be partitioned into a separate publication focused solely on sex differences.

Author response: We appreciate the reviewer's thoughtful suggestion. Although we included participants of both sexes for greater generalizability of the results, the primary goal of our study was not to evaluate a potential sex-specific difference in mitochondrial affinity for O_2, and the study is therefore not powered to address this question. We agree with the reviewer and confirm that we have no intention to publish a separate manuscript focusing on sex-specific outcomes from this dataset.

Based on your suggestion, we conducted a secondary analysis to assess the potential influence of sex on our primary outcomes. This analysis did not reveal any appreciable sex differences within our small sample. While we acknowledge that sex-based differences may influence muscle metabolism and mitochondrial function, most studies have failed to detect such differences. For instance, Kent-Braun et al. (2002) found no significant sex-related variation in muscle oxidative capacity assessed by PCr recovery kinetics in young adults, and Russ et al. (2005) similarly documented no sex differences in mitochondrial oxidative capacity as indicated by similar PCr recovery kinetics between men and women (Kent-Braun et al., 2002; Russ et al., 2005). Recently, Bailleul et al. (2025) reported no sex effect on fiber-specific oxidative capacity (Bailleul et al., 2025). These findings are consistent with our secondary analysis, which did not reveal sex differences in mitochondrial P50.

Based on your comment, this paragraph has been added into a new section labeled "experimental consideration" (Line 420-429).

Bailleul, C., Hodson, N. Sawan, S., Kumbhare, D., Moore, D., Gillen, J. (2025). The influence of sex on fiber-specific indices of oxidative capacity in human skeletal muscle. American Journal of Physiology - Regulatory Integrative and Comparative Physiology, 329(1), R70-R80.

Kent-Braun, J., Ng, A.V., Doyle, J.W., Towse, T.F. (2002). Human skeletal muscle responses vary with age and gender during fatigue due to incremental isometric exercise. Journal of Applied Physiology, 93, 1813-1826.

Russ, D.W., Lanza, I.R., Rothman, D., Kent-Braun, J. (2005). Sex differences in glycolysis during brief, intense isometric contractions. Muscle and Nerve, 32(5), 647-655.

Reviewer 1 (Minor): The opening paragraph provides a useful overview of the oxygen transport cascade; however, several aspects would benefit from clearer framing and greater physiological precision. As written, the paragraph presents specific partial pressure values (e.g., ambient, tissue, and intramuscular PO_2) and implies that O_2 transport and respiratory flux are occurring primarily in skeletal muscle. These assumptions should be stated more explicitly and contextualized, especially given that the present study investigates resting muscle, where skeletal muscle accounts for only a modest fraction of whole-body oxygen consumption. Clarifying that the cited PO_2 values represent typical approximations, rather than fixed physiological constants, would avoid unintentionally overgeneralizing conditions that vary with environment, measurement technique, and metabolic state. In addition, tightening the biochemical language would improve accuracy. For example, mitochondria do not "produce" energy; rather, they transduce the free energy from substrate oxidation into ATP through oxidative phosphorylation. Revising the phrasing accordingly (e.g., referring specifically to ATP production or energy transduction) would align the introduction with contemporary biochemical understanding. Overall, refining the assumptions and terminology in this initial paragraph will bring it into better alignment with the scientific precision demonstrated throughout the remainder of the Intro.

Author response: We thank the reviewer for their suggestions to improve the physiological precision and biochemical clarity of the introduction. Accordingly, we added the following sentences within the first paragraph of the introduction. Additionally, we replaced the term “produced” with “transduced” to more accurately reflect the role of mitochondria in energy metabolism.

Line (71-73): “Of note, such values represent typical approximations that can vary substantially depending on the environments and the metabolic state as the resting skeletal muscle only accounts for a modest fraction of whole-body oxygen consumption.”

Line (77): The appropriate term was adjusted from “produced” → “transduced”

Review 1 (Minor): For both Vmax and P50, the presentation of the results would benefit from clearer statistical formatting or explanation. The manuscript reports values such as "Vmax = 0.20 {plus minus} 0.06 mM·min^{-1} with the 95% CI of 0.17 {plus minus} 0.05 to 0.23 {plus minus} 0.09 mM·min^{-1}" and "P50 = 0.74 {plus minus} 0.55 mmHg with the 95% CI of 0.42 {plus minus} 0.30 to 0.87 {plus minus} 0.74 mmHg." As written, it is unclear whether the {plus minus} values accompanying the CI bounds represent inter-individual variability in the limits, or whether they denote another statistical quantity. Conventionally, the 95% CI for the group mean is expressed simply as two values (e.g., 0.17-0.23), whereas the SD or SEM is presented separately as mean {plus minus} SD (or SEM).

Author response: We thank the reviewer for highlighting this point regarding the statistical clarity. To clarify, we performed nonlinear curve fitting on each individual's data to estimate P50 and Vmax, and we then reported the group mean ± SD of those individual parameter estimates. For the 95% confidence intervals, we computed them for each participant's fitted parameters and then reported the group-level CI bounds as the mean ± SD of the individual CI limits. In response to this comment, we have revised both the Statistical Analysis **(Line: 190-194)** and Results sections to more clearly explain our approach and improve the precision of our formatting. Specifically, we now clarify that the group-level CI bounds reflect the average of individual confidence limits. We hope this explanation resolves the ambiguity and enhances the interpretability of the reported statistics.

Reviewer 1 (minor): Figure 2: It would be helpful to distinguish the VO_2-PO_2 curves for male and female participants in the lower right panel (e.g., by using solid vs. dashed lines) so that readers can more easily see whether sex-specific patterns are present. In addition, please confirm that all 11 participants are represented. The current panel appears to show 10 curves, although two may be superimposed.

Author response: We confirm that all 11 participants are represented in the lower-right panel of Figure 2. Two participants exhibited highly similar VO_2–PO_2 responses, resulting in visually overlapping curves. Regarding the suggestion to distinguish curves by sex, we recognize the potential value of this approach for visual interpretation. However, given that our secondary analysis did not reveal appreciable sex differences in P50 or Vmax, displaying sex-specific lines might inadvertently overstate differences that were not statistically supported in this sample. To preserve the clarity of the figure, we have therefore decided to keep the figure as is. However, if even after our explanation the reviewer continues to feel strongly about this issue, we will be happy to reconsider this visualization to identify male and female participants in the inset.

Reviewer 1 (minor): When referencing prior reports of P50 values from ex vivo preparations, could you please specify which studies employed hyperoxygenated chambers versus ambient (room air) saturation? This distinction is important for contextualizing differences in apparent oxygen affinity. You may also consider citing recent work from a lab I know you are familiar (PMID: 40803624), which discusses/details these importance of these methodological considerations.

Author Response: We agree that the chamber oxygenation strategy should be specified when comparing ex vivo P50 values. We revised the Discussion to (i) explicitly distinguish studies initiated near air saturation versus those using hyperoxygenated chambers when this information was reported **(Line: 256-270)**, and (ii) note that many ex vivo P_{50} datasets were generated in sealed/closed preparations in which PO_2 was allowed to decline during measurement. We also added the suggested citation (PMID: 40803624).

Line (262-270): For instance, the cell-suspension P_{50} estimates (Wilson et al., 1977; Robiolio et al., 1989; Rumsey et al., 1990) were obtained in sealed cuvette/syringe preparations, in which PO_2 was allowed to decline progressively from near air saturation (reported as ~160 mmHg in Robiolio et al., 1989), and subsequently reset by the addition of H_2O_2 in the presence of catalase, rather than maintaining a hyperoxygenated chamber. Similarly, for isolated mitochondria, where oxygenation conditions were explicitly described, measurements were conducted at ambient or sub-ambient

oxygenation (Barzu and St-Pierre), whereas Cardinale et al. (2019) used hyperoxygenated chambers to minimize diffusion limitation.

Reviewer 1 (minor): In the final sentence of the first discussion paragraph, you state, "Yet, O_2 levels may still exert some control over ATP production under some specific conditions in vivo characterized by critically low intracellular PO_2." As currently placed, this assertion feels somewhat premature, as the manuscript does not experimentally examine the influence of altered PO_2 on respiratory flux or ATP production. I recommend removing this statement here and just focusing this discussion for later, as was included.

Author Response: Thank you for pointing this out. We agree that the original placement of this sentence may have implied a stronger causal interpretation than our data directly support at that stage of the discussion. Accordingly, we have removed this sentence from the end of the first paragraph.

Reviewer 1 (minor): Your comparison to ex vivo preparations requires some clarification. The manuscript suggests that 'closed-chamber respirometry minimizes O_2 gradients', but this is not consistent with established methodological work (Donnelly C, Schmitt S, Cecatto C, Cardoso LHD, Komlódi T, Place N, Kayser B, Gnaiger E (2022) The ABC of hypoxia - what is the norm. Bioenerg Commun 2022.12.v2. https://doi.org/10.26124/bec:2022-0012.v2). Closed-chamber systems inherently develop O_2 gradients, which is the very reason laboratories should employ hyperoxygenated environments (and why I am asking you to make this distinction across applicable references included throughout your paper). Hyperoxygenation mitigates, but does not eliminate, O_2 gradients, and it introduces its own considerations (e.g., increased ROS exposure). As currently written, the statement could inadvertently imply that closed-chamber designs reduce O_2-dependence without qualification, which may unintentionally validate suboptimal approaches in labs that do not hyperoxygenate. I suggest revising this portion of the discussion to more accurately reflect the limitations of closed-chamber respirometry or providing references to support the present interpretation.

Author Response: We fully concur with the reviewer that closed-chamber respirometers do not completely eliminate O_2 gradients. This statement was meant to indicate that we focused on *ex vivo* studies using closed-chamber respirometers, which are more suited for those types of experiments at low O_2 concentration than other instruments containing both gas and liquid medium (Cole et al., 1982; Gnaiger et al., 1995; Wittenberg & Wittenberg, 1985). The latter type of instruments results in a steep O_2 gradient and more heterogeneous PO_2 across the chamber, which can confound the quantification of the P_{50}.

Your point is, however, well-taken. We revised the paragraph of this Discussion section (and edited the wording around the ex vivo comparison) and Figure legend 3 to explicitly state that diffusion gradients can develop in sealed systems, especially at higher respiratory flux, and that hyperoxygenation and/or correction approaches are commonly used to mitigate diffusion limitation.

Line (256-268): "Of note, most ex vivo estimates reported here were obtained from closed-chamber respirometers, enabling more accurate assessment of mitochondrial respiration than 2-phase system instruments, for which more heterogeneous PO_2 introduces some errors in the estimation of the P_{50} at low PO_2 in the early studies on this topic (Cole et al., 1982; Gnaiger et al.,

1995; Wittenberg & Wittenbergs, 1985). However, closed-chamber respirometers still inherently exhibit O_2 gradients, especially at higher respiratory flux (Donnelly et al., 2022), and various oxygenation strategies have been used across studies to attenuate such effects. For instance, cell-suspension P_{50} estimates were obtained in sealed cuvette/syringe preparations, in which PO_2 was allowed to decline progressively near air saturation (Robiolio et al., 1989; Rumsey et al., 1990; Wilson et al., 1977). For isolated mitochondria, measurements were generally conducted at ambient or sub-ambient oxygenation (Barzu & Satre, 1970; St-pierre et al., 2000), except for the study by Cardinale et al. (2018), which hyperoxygenated the chambers. Despite this methodological difference, the resulting P_{50} values remained within a tight range, underscoring the high intrinsic O_2 affinity of cytochrome c oxidase (COX). Overall, the majority of the literature, including our study, supports an apparent mitochondrial P_{50} for O_2 above 0.1 and below 1.7 mmHg across *in vitro* and *in vivo* preparations, which also supports the concept that intramyocellular PO_2 around the mitochondrial network is homogeneous (Clark et al., 1987).

Reviewer 1 (Minor): Paragraph 259-273: The section discussing how mitochondrial content may influence the observed Vmax and P50 values is particularly interesting, and I encourage the authors to expand this aspect of the discussion. A more concise summary of the Gayeski et al. (1987) findings would improve readability, while allowing more space to integrate your results with contemporary understanding of mitochondrial distribution/heterogeneity and respiratory control. Additionally, given that the current data are derived from a single muscle group at rest, it would be helpful to briefly elaborate on whether similar relationships would be expected in other skeletal muscles or even in non-muscle tissues (i.e., liver, which can become much more hypoxic in specific areas than muscle), to further contextualize the broader relevance of your findings.

Author Response: We agree and have revised this section to improve readability and expand the discussion of how mitochondrial content may influence the observed Vmax and apparent P50. Specifically, we (i) condensed the description of Gayeski et al. (1987) to the key finding and the most relevant methodological constraints (ii) expanded the discussion of mitochondrial volume/content and respiratory control at rest by integrating evidence from endurance-trained skeletal muscle showing increased oxidative flux without higher resting ATP synthesis, consistent with greater uncoupling/proton leak under low resting ADP (Befroy et al., 2008) as well as endurance-training related increases in fatty-acid stimulated proton leak that prevents excessive ROS formation (Tonkonogi et al., 2000), and (iii) added a brief statement that similar qualitative relationships may be expected across other skeletal muscles and non-muscle tissues, but that the quantitative relationship between Vmax and apparent P50 may differ depending on mitochondrial distribution/heterogeneity and local oxygenation (e.g., microregional hypoxia in liver). Thank you for prompting these additions to the manuscript (Line: 275-302).

Reviewer 1 (Minor): Expanding the discussion to include the regulatory role of myoglobin in shaping intracellular PO_2 dynamics (e.g., PMID: 30335576) would add valuable biological context, particularly for interpreting how your resting measurements relate to conditions of increased metabolic demand, such as exercise. You reference this work toward the end of the discussion, which is an excellent start; extending this section slightly would help readers better understand how myoglobin-mediated buffering and facilitated diffusion influence mitochondrial PO_2 and respiratory control in vivo.

Author Response: We thank the reviewer for this suggestion. We expanded the Discussion in the section linking resting measurements to conditions of increased metabolic demand to more explicitly describe the role of myoglobin in shaping intracellular PO_2 dynamics. Specifically, we added a brief text explaining myoglobin-mediated buffering and facilitated diffusion and how these mechanisms may influence mitochondrial PO_2 and respiratory control during heavy exercise (Clanton, 2019; PMID: 30335576).

Line (365-372): "In the low-PO_2 operating range (Figure 2), myoglobin (Mb) may influence intracellular PO_2 dynamics beyond serving as a O_2 transporter. Because Mb desaturates steeply as PO_2 approaches its P_{50} (~2.2 mmHg), it buffers rapid changes in intracellular PO_2 and facilitates intracellular O_2 transport when diffusion gradients increase at high O_2 flux. Clanton (2019) conceptualized Mb as part of an integrated 'power-grid' with the mitochondrial reticulum, proposing that Mb-mediated buffering/facilitated diffusion (and PO_2-dependent NO interactions) can dampen intracellular PO_2 heterogeneity and modulate respiratory control during heavy exercise."

References

Connett, R. J., Honig, C. R., J Gayeski, T. E., & Brooks, G. A. (1990). *Defining hypoxia: a systems view of VO2, glycolysis, energetics, and intracellular PO2*. *68*(3), 833–842.

Grassi, B., Poole, D. C., Richardson, R. S., Knight, D. R., Erickson, B. K., & Wagner, P. D. (1996). Muscle O2 uptake kinetics in humans: Implications for metabolic control. *Journal of Applied Physiology*, *80*(3), 988–998. https://doi.org/10.1152/jappl.1996.80.3.988

Haseler, L. J., Hogan, M. C., & Richardson, R. S. (1999). Skeletal muscle phosphocreatine recovery in exercise-trained humans is dependent on O2 availability. *J. Appl. Physiol*, *86*(6), 2013–2018. http://www.jap.org

Haseler, L. J., Lin, A. P., & Richardson, R. S. (2004). Skeletal muscle oxidative metabolism in sedentary humans: 31 P-MRS assessment of O 2 supply and demand limitations. *J Appl Physiol*, *97*, 1077–1081. https://doi.org/10.1152/japplphysiol

Haseler, L. J., Richardson, R. S., Videen, J. S., & Hogan, M. C. (1998). Phosphocreatine hydrolysis during submaximal exercise: the effect of FIO2. *J. Appl. Physiol*, *85*(4), 1457–1463. http://www.jap.org

Hogan, M. C., Nioka, S., Brechue, W., & Chance, B. (1992). A 31P-NMR study of tissue respiration in working dog muscle during reduced O2 delivery conditions. *Journal of Applied Physiology*, *73*(4), 1662–1670.

Hogan, M. C., Richardson, R. S., & Haseler, L. J. (1999). Human muscle performance and PCr hydrolysis with varied inspired oxygen fractions: A 31P-MRS study. *Journal of Applied Physiology*, *86*(4), 1367–1373. https://doi.org/10.1152/jappl.1999.86.4.1367

Hughson, R. L. (2009). Oxygen uptake kinetics: Historical perspective and future directions. *Applied Physiology, Nutrition and Metabolism*, *34*(5), 840–850. https://doi.org/10.1139/H09-088

Jones, A. M., & Poole, D. C. (2005). Oxygen uptake dynamics: From muscle to mouth - An introduction to the symposium. *Medicine and Science in Sports and Exercise*, *37*(9), 1542–1550. https://doi.org/10.1249/01.mss.0000177466.01232.7e

Korzeniewski, B. (1998). Regulation of ATP supply during muscle contraction : theoretical studies. In *Biochem. J* (Vol. 330).

Korzeniewski, B. (2007). Regulation of oxidative phosphorylation through parallel activation. *Biophysical Chemistry*, *129*(2–3), 93–110. https://doi.org/10.1016/j.bpc.2007.05.013

Korzeniewski, B., & Rossiter, H. B. (2015). Each-step activation of oxidative phosphorylation is necessary to explain muscle metabolic kinetic responses to exercise and recovery in humans. *Journal of Physiology*, *593*(24), 5255–5268. https://doi.org/10.1113/JP271299

Layec, G., Haseler, L. J., & Richardson, R. S. (2013). Reduced muscle oxidative capacity is independent of O2 availability in elderly people. *Age*, *35*(4), 1183–1192. https://doi.org/10.1007/s11357-012-9442-6

Layec, G., Haseler, L. J., Trinity, J. D., Hart, C. R., Liu, X., Fur, Y. Le, Jeong, E. K., & Richardson, R. S. (2013). Mitochondrial function and increased convective O2 transport: Implications for the assessment of mitochondrial respiration in vivo. *Journal of Applied Physiology*, *115*(6), 803–811. https://doi.org/10.1152/japplphysiol.00257.2013

Layec, G., Trinity, J. D., Hart, C. R., Le fur, Y., Soresen, J. R., Jeong, E.-K., & Richardson, R. S. (2017). Evidence of metabolic reserve in skeletal muscel of elderly people. *Aging*, *9*(1), 52–67.

Poole, D. C., Behnke, B. J., & Musch, T. I. (2021). The role of vascular function on exercise capacity in health and disease. *Journal of Physiology*, *599*(3), 889–910. https://doi.org/10.1113/JP278931

Poole, D. C., & Jones, A. M. (2012). Oxygen uptake kinetics. *Comprehensive Physiology*, *2*(2), 933–996. https://doi.org/10.1002/cphy.c100072

Richardson, R. S., Leigh, J. S., Wagner, P. D., & Noyszewski, E. A. (1999). Cellular PO2 as a determinant of maximal mitochondrial O2 consumption in trained human skeletal muscle. *Journal of Applied Physiology*, *87*(1), 325–331. http://www.jap.org

Richardson, R. S., Noyszewski, E. A., Kendrick, K. F., Leigh, J. S., & Wagner, P. D. (1995). Myoglobin 02 Desaturation during Exercise Evidence of Limited 02 Transport. *J. Clin. Invest*, *96*, 1916–1926.

Richardson, R. S., Wary, C., Wray, D. W., Hoff, J., Rossiter, H. B., Layec, G., & Carlier, P. G. (2015). MRS evidence of adequate O2 supply in human skeletal muscle at the onset of exercise. *Medicine and Science in Sports and Exercise*, *47*(11), 2299–2307. https://doi.org/10.1249/MSS.0000000000000675

Tschakovsky, M. E., Hughson, R. L., Jones, A. M., Berger, N. J. A., Wilkerson, D. P., Roberts, C. L., Kowalchuk, M., Gurd, B. J., Peters, S. J., Heigenhauser, G. J. F., Leblanc, P. J., Doherty, T. J., Paterson, D. H., Forbes, S. C., Kowalchuk, J. M., Thompson, R. T., Marsh, G. D., Berger, N. J., Fulford, J., … Rossiter, H. B. (1999). Interaction of factors determining oxygen uptakeat the onset

of exercise. *J Appl Physiol J. Physiol. J Appl Physiol Am J Physiol Regulatory Integrative Comp Physiol J. Physiol. Journal of Applied Physiology*, *86*(5), 1432–1441.

Wagner, P. D. (1996). A theoretical analysis of factors determining Vo2MAX at sea level and altitude. *Respiration Physiology*, *106*, 329–343.

Weibel, E. R., Taylor, C. R., & Hoppeler, H. (1991). The concept of symmorphosis: A testable hypothesis of structure-function relationship. *Proc. Natl. Acad. Sci. USA*, *88*, 10357–10361.

Wüst, R. C. I., Grassi, B., Hogan, M. C., Howlett, R. A., Gladden, L. B., & Rossiter, H. B. (2011). Kinetic control of oxygen consumption during contractions in self-perfused skeletal muscle. *Journal of Physiology*, *589*(16), 3995–4009. https://doi.org/10.1113/jphysiol.2010.203422

Reviewer #2:

Reviewer 2 comment: First, all of the results in the study appear to be calculated from the data shown in Figure 1B. If this is the case, wouldn't the correlations seen in Figure 4 be expected? Could the authors confirm the independence of their two main variables?

Author Response: While both Vmax and P50 are derived from the Michaelis–Menten VO2-iPO2 relationship, they represent independent parameters of the model rather than dependent quantities. Each participant's curve was fit using nonlinear least-squares regression to simultaneously estimate Vss (the asymptotic maximum) and P50 (the PO2 at half-maximal VO2). The observed correlation across participants therefore does not arise from shared calculation steps within individuals, but rather reflects inter-individual variation in these independently fitted parameters. To further test this, we performed the correlation using log-transformed variables, which preserved both the significance and slope of the relationship ($r = 0.83$, $p < 0.01$; Figure 4B), indicating that the association is not a mathematical artifact of the curve fitting. This suggests a genuine physiological coupling between mitochondrial respiratory flux and O_2 affinity under resting conditions in vivo.

Reviewer 2 Comment: Second, the paper uses an assumed value of 0.5 mM for [Mb]. When calculating and comparing rate constants, it is not uncommon to assume a constant and uniform concentration. However, formally this leaves the resulting calculations with a maximum resolution of one significant digit. The importance of this assumption needs to be clarified in the discussion and limitations to the paper. As a suggestion, could the authors normalize their [Mb] values? Given the assumption that [ATP] values are tightly regulated, especially at rest, could the authors normalize their [Mb] values to [b-ATP] values? I am aware that 31P MRS was not performed in this study, but the authors should have this data from previous studies. Assuming a mean value of 0.5 mM and normalizing might add precision to the measurements.

Author Response: We concur with the reviewer that the absolute quantification of the Mb signal is an important topic (as for any MRS signal), and is not trivial to accurately perform.

In our approach, the assumed [Mb] (0.5 mM) is used to convert the change in deoxy-myoglobin signal during ischemia into an absolute rate of VO2 in concentration units. Thus, it primarily affects the absolute scaling of VO_2 and the resulting Vss. In contrast, mitochondrial P50 is determined by the shape of the VO_2-iPO_2 relationship (i.e., the iPO_2 at which VO_2 reaches 50% of Vss) and is therefore expected to be insensitive to a scaling factor applied to VO_2. In other words, if the true [Mb] differed from 0.5 mM, VO_2 and Vss would shift proportionally, whereas P_{50} would remain essentially unchanged.

Based on your comment, we decided to confirm this by evaluating the impact of the assumed [Mb] on our key outcome, we performed a sensitivity analysis in which VO_2 was rescaled using a range of [Mb] values (e.g., 0.2 and 0.8 mM vs. 0.5 mM) and the VO_2-iPO_2 relationship was fit. As expected, Vmax changed proportionally with [Mb], whereas the estimated P_{50} was essentially unchanged. This is illustrated in the sensitivity simulation figure below, where the assumed [Mb] proportionally rescales VO2 and Vmax, while the corresponding P50 remains unchanged. These analyses support that our primary conclusions, namely the very low in vivo mitochondrial P_{50} and the delineation of O_2-independent versus O_2-sensitive operating ranges, are robust and independent from the [Mb] assumption.

Although the assumption about [Mb] does not alter the estimation of the P50, we did consider an absolute quantification of Mb using by normalizing its peak to the water signal. However, this approach also suffers of flaws. Normalization to the water signal requires additional acquisitions and corrections (e.g., separate receiver gain/dynamic range handling due to the very large water signal), and still requires an assumption about the water content. Each of these steps introduce additional variance and will not necessarily improve the reliability of the VO_2-iPO_2 curve-shape parameters that determine P_{50}.

Regarding the normalization to β-ATP, we did not acquire ^{31}P-MRS in the present study. Therefore, using β-ATP values from separate cohorts would not provide subject-specific normalization and could introduce additional errors due to differences in acquisition conditions (coil-signal sensitivity, difference in sensitivity channels to ^{31}P and ^{1}H).

Based on the limitations of the different approaches considered, the absolute quantification of the Mb signal assuming [Mb] is 0.5 mM still provides greater accuracy.

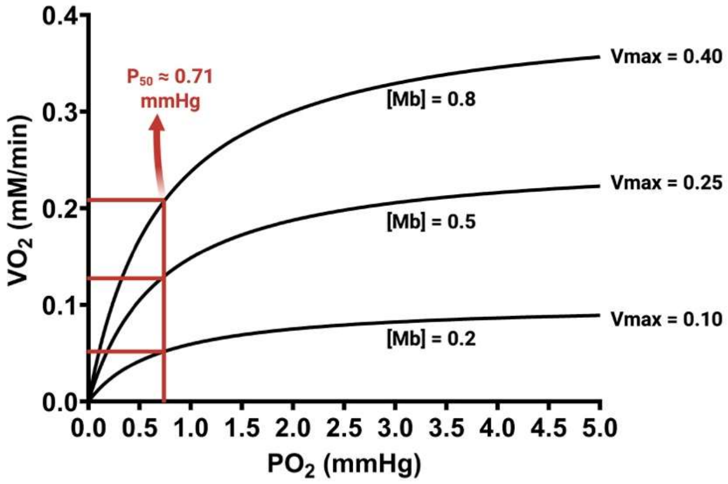

Reviewer 2 Comment: Vmax is used to represent the highest metabolic rate under resting conditions. Vmax is typically used to represent the highest enzymatic rate, and this is not achieved during resting conditions. This seems confusing and it would be helpful to the reader if a different terms was used.

Author Response: Thank you for raising this concern. We agree that the term *Vmax* can be confusing in this context. In our analysis, this parameter represents the plateau (asymptotic) of the fitted VO_2–iPO_2 relationship under resting conditions, not a whole-muscle maximal metabolic rate. Based on your comment, we replaced *Vmax* throughout with V Steady-State (**V_{ss})** and updated the text and figure labels accordingly. We modified the following sentences in the method section

Line (185-187): Where VO_2 denotes the measured VO_2 in the skeletal muscle, V_{ss} is the steady-state in O_2 consumption rate estimated from the VO_2-iPO_2 fit under resting conditions, and P_{50} is the partial pressure of O_2 at which O_2 consumption reaches half of V_{ss}.

Thank you.

Reviewer 2 Comment: Line 172. It seems confusing to refer to the 8 minute occlusion period. Mb VO2 was actually calculated from the first minute or second minute of the occlusion period?

Author Response: We thank the reviewer for pointing out this potential ambiguity. The dMb-derived VO_2 was calculated from the initial phase of the dMb desaturation curve, corresponding approximately to the initial 4-5 minutes of the entire occlusion period, when intracellular oxygenation declined (dMb peak increased). The remainder of the 8-minute occlusion ensured full deoxygenation of myoglobin, which was used to normalize the dMb signal to 100% desaturation for calibration. To clarify this point, we have revised the text accordingly.

Line (174-176): Mb-derived VO_2 was calculated from the **initial portion** of the dMb desaturation time course (typically within the first ~4-5 minutes of occlusions), whereas the full 8-minute period was used to reach the end-ischemic plateau for normalization.

Reviewer 2 Comment: Line 178. Minor point, but 39 degrees C is 102.2 Fahrenheit. Muscle temperature in the area under the MRS coil is most likely closer to 32-34 degrees C. (assuming skin is 30 degrees and the MRS data is collected several cm deep into the muscle. This does not account for the wrap of the coil keeping the limb warm or the temperature of the room (which is often cool).

Author Response: We thank the reviewer for bringing up this important point about the temperature dependency of the myoglobin P_{50} as our value were interpolated from the exercising muscle values (Richardson et al., 1995, 2006). Your comment made us realize that this value was inadequate to the present conditions. We conducted a literature search, which indicated that muscle temperature in the leg appears to be closer to ~35-36°C (Kenny et al., 2003) at rest in a thermoneutral environment. At such temperature, the Mb P_{50} is then closer to 2.2 mmHg (Schenkman et al., 1997) **(Line: 181-182)**. We have therefore reconsidered our entire analysis in light of this new value, and our mean mitochondrial P50 estimate is now ~0.50 mmHg. Thank you for pointing out this inaccuracy.

Reviewer 2 Comment: Figure 5. This is a nice graph. But there maybe some conceptual issues. Capillary values might be 'end capillary' as the fall in oxygen levels reflects the flux of oxygen from the vessels into the tissue. End capillary is after this exchange has occurred. Mitochondrial values might be listed in addition to the P50 values. Mitochondrial values might be expected to be higher than the P50. The P50 is not the actual oxygen pressure, but the value at which metabolism is half resting.

Author Response: Thank you for this important and helpful comment. We agree that the original version of Figure 5 could be interpreted as conflating the actual physical oxygen partial pressure along the O_2 cascade with the mitochondrial P_{50} value, which is a kinetic parameter. Therefore, we revised Figure 5 to improve the conceptual distinction and address your concerns by relabeling capillary PO_2 as "End-capillary" to better reflect oxygen tension, and by showing the mitochondrial portion as a range/annotated region rather than implying that P50 itself represents the actual mitochondrial PO_2. We measured P_{50} in the present study as a mitochondrial oxygen-affinity parameter (i.e., the PO_2 at which mitochondrial respiration is 50% of maximal). In addition, we now indicate the approximate PO_2 value (~4.5 mmHg) corresponding to 10%

inhibition of respiration to provide the physiological range of O2 dependence beyond P_{50} alone. We also clarified in the figure legend that P_{50} mito is a fitted kinetic parameter.

Dear Dr Layec,

Re: JP-RP-2026-290548R1 "**In vivo Quantification of Mitochondrial O_2 Affinity in Human Skeletal Muscle Using ^{1}H-MR Spectroscopy of Deoxy-Myoglobin**" by Muhammet Enes Erol, Alexs A Matias, Corinna F Serviente, Stephen T Decker, Rajakumar Nagarajan, Saadallah Ramadan, Yann Le Fur, Song Young Park, and Gwenael Layec

We are pleased to tell you that your paper has been accepted for publication in The Journal of Physiology.

The last Word (or similar) version of the manuscript provided will be used by the Production Editor to prepare your proof. When this is ready you will receive an email containing a link to Wiley's Online Proofing System. The proof should be thoroughly checked and corrected as promptly as possible.

Authors should note that it is too late at this point to offer corrections prior to proofreading. Major corrections at proof stage, such as changes to figures, will be referred to the Editors for approval before they can be incorporated. Only minor changes, such as to style and consistency, should be made at proof stage. Changes that need to be made after proof stage will usually require a formal correction notice.

All queries at proof stage should be sent to: TJP@wiley.com.

Following copyediting and typesetting the accepted version will be published online.

Yours sincerely,

Karyn Hamilton
Senior Editor
The Journal of Physiology

IMPORTANT POINTS TO NOTE FOLLOWING ACCEPTANCE OF YOUR PAPER:

• IMPORTANT NOTICE ABOUT OPEN ACCESS: To assist authors whose funding agencies mandate immediate public access to published research findings, The Journal of Physiology allows authors to pay an Open Access (OA) fee to have their papers made freely available immediately on publication.

The Corresponding Author will receive an email from Wiley with details on how to register or log in to Wiley Authors where you will be able to place an order.

• You can check if your funder or institution has a Wiley Open Access Account here:
https://authors.wiley.com/author-resources/Journal-Authors/open-access/author-compliance-tool.html

• TRANSPARENT PEER REVIEW POLICY: To improve the transparency of its peer review process, The Journal of Physiology publishes online as supporting information the peer review history of all articles accepted for publication. Readers will have access to decision letters, including Editors' comments and referee reports, for each version of the manuscript as well as any author responses to peer review comments. Referees can decide whether or not they wish to be named on the peer review history document.

• You can help your research get the attention it deserves! Check out Wiley's free Promotion Guide for best-practice recommendations for promoting your work at: www.wileyauthors.com/eeo/guide. You can learn more about Wiley Editing Services which offers professional video, design, and writing services to create shareable video abstracts, infographics, conference posters, lay summaries, and research news stories for your research at: www.wileyauthors.com/eeo/promotion.

• If you would like to receive our 'Research Roundup', a monthly newsletter highlighting the cutting-edge research published in The Physiological Society's family of journals (The Journal of Physiology, Experimental Physiology, Physiological Reports, The Journal of Nutritional Physiology and The Journal of Precision Medicine: Health and Disease), please click this link, fill in your name and email address and select 'Research Roundup':
https://www.physoc.org/journals-and-media/membernews

EDITOR COMMENTS

Reviewing Editor:

Thank you for your thorough revision, both reviewers indicate that their concerns have been sufficiently addressed.

Senior Editor:

Thank you for submitting your revised manuscript. We are pleased to accept it for publication in The Journal of Physiology. Thank you for your interest in The Journal and Congratulations!

REFEREE COMMENTS

Referee #1:

The authors have addressed my previous comments thoroughly and have made meaningful revisions to improve the clarity, context, and overall presentation of the manuscript. While the statistical approach used to report confidence intervals remains somewhat unconventional, the authors have now clearly explained their methodology, which resolves my initial concerns regarding interpretability. Overall, I am satisfied with the revisions and have no further concerns.

Referee #2:

The authors have done an excellent job of responding to the prior review. I have no further suggestions for the authors.

****
